# Synthetic Routes to Silsesquioxane-Based Systems as Photoactive Materials and Their Precursors

**DOI:** 10.3390/polym11030504

**Published:** 2019-03-16

**Authors:** Beata Dudziec, Patrycja Żak, Bogdan Marciniec

**Affiliations:** 1 Department of Organometallic Chemistry, Faculty of Chemistry, Adam Mickiewicz University in Poznan, Umultowska 89B, 61-614 Poznan, Poland; bogdan.marciniec@amu.edu.pl; 2 Centre for Advanced Technologies, Adam Mickiewicz University in Poznan, Umultowska 89C, 61-614 Poznan, Poland

**Keywords:** silsesquioxanes, optoelectronics, OLEDs

## Abstract

Over the past two decades, organic optoelectronic materials have been considered very promising. The attractiveness of this group of compounds, regardless of their undisputable application potential, lies in the possibility of their use in the construction of organic–inorganic hybrid materials. This class of frameworks also considers nanostructural polyhedral oligomeric silsesquioxanes (POSSs) with “organic coronae” and precisely defined organic architectures between dispersed rigid silica cores. A significant number of papers on the design and development of POSS-based organic optoelectronic as well as photoluminescent (PL) materials have been published recently. In view of the scientific literature abounding with numerous examples of their application (i.e., as OLEDs), the aim of this review is to present efficient synthetic pathways leading to the formation of nanocomposite materials based on silsesquioxane systems that contain organic chromophores of complex nature. A summary of stoichiometric and predominantly catalytic methods for these silsesquioxane-based systems to be applied in the construction of photoactive materials or their precursors is given.

## 1. Introduction

**Figure 1:**
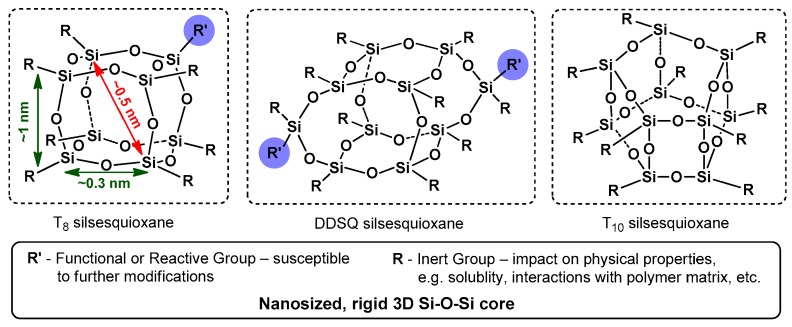
The hybrid (i.e., organic–inorganic) nanostructures of respective cubic T_8_, DDSQ and T_10_ silsesquioxanes.

Functionalized polyhedral oligomeric silsesquioxanes (POSSs) [1] with a general (RSiO_3/2_)_n_ formula are inorganic–organic systems composed of a well-defined Si–O–Si core with organic moieties that have become the representative building blocks for hybrid materials (Figure 1).

Due to their finely tunable physical and chemical properties, POSSs meet the requirements of both science and industry [2,3]. The silsesquioxane family group contains a small number of well-defined 3D structures. The most recognizable and best known of this is cubic T_8_ with either one or eight functional group derivatives [3]. Recently, the so-called double-decker-type silsesquioxane (DDSQ) structure, which differs from the symmetric, cubic one, and features opened (M_4_T_8_ = DDSQ-4OSi [4]) or closed (D_2_T_8_ = DDSQ-2Si [5]) frameworks with either two or four reactive moieties, has also gained respective interest [6]. The physicochemical properties of functionalized silsesquioxanes are a particularly important aspect of the research on their synthesis. This is due to their chemical and spatial structure resulting in a hybridic (i.e., organic–inorganic) nature. The presence of a regular element of the Si–O–Si framework corresponds with the silica architecture places silsesquioxanes as fillers (nanofillers) with a perfectly defined structure and nanometric dimensions for polymers modifiers [7]. The well-defined sizes of their molecules, and the presence of different amount and type of functional groups at the POSS core, allows their use in the precise deposition/embedding in the polymer matrix and obtain composite materials with unique properties [2,3,8,9]. The obtained organic–inorganic hybrid materials are characterized by a whole range of interesting physicochemical features (e.g., thermal, mechanical, optical and chemical). This is related to their solubility change, increase in decomposition and glass transition temperatures, improvement of dielectric properties, reduction of heat transfer coefficients, and improvement of oxidation and fire resistance, as well as their impact on the hardness of obtained materials [2,8,10,11]. Due to these aspects, functional silsesquioxanes possess great application potential [2] and could be applied as nanofillers and components of polymers [8,11], building blocks and synthons for a variety of advanced materials with tailored properties [12,13], such as silica models [14], (super)hydrophobic coatings [2], dendrimers and metal carriers. They were applied as immobilizing phase in catalysts [13,15], medicine (e.g., as drugs carriers, components of artificial tissues or in stomatology [16,17]) and opto- and electroluminescent (EL) materials [18]. 

Organic light emitting diodes (OLEDs) are a highly targeted area of technology because of their expected utility in flat panel displays. There have been discussions over the past decade concerning the family of small molecules and polymers that is best suited for OLEDs [18,19,20,21,22,23]. Small molecules can be highly purified and vacuum-deposited in multi-layer stacks, which is important both for display lifetime and efficiency. However, vacuum deposition techniques are expensive and limit the practical application, which causes problems achieving full colour displays at high volume. On the other hand, polymers are generally not as pure as small molecules, but can access larger display sizes and full colour at less substantial expense via solution-based deposition techniques.

Nanocomposite materials based on a silsesquioxane architecture that combines the advantages of both small-molecule and organic polymer fragments have been applied to OLEDs. They were introduced by Sellinger et al. in 2003 [24], with the compounds containing a spherical ‘‘silica’’ core with a hole-transporting functionalized periphery (chromophores). The resulting materials have offered numerous advantages for OLEDs, including amorphous properties enhancing thermal resistance (i.e., high glass-transition temperatures (*T*_g_)) and stabilized colour at higher temperatures, as well as low polydispersity, solubility, and high purity. Silsesquioxanes also help to reduce (prevent) the aggregation of chromophores that are susceptible to the π–π intermolecular interaction (aggregation) leading to quenching the fluorescence. In general, the key aspect of the POSS-based OLEDs is to use them as scaffolds for organic chromophores of complex nature, bearing in mind that the characteristic parameters for OLEDs include wavelength (color of light emitted), luminance (a brightness greater than 10,000 cd m^−2^ is desirable) and external quantum efficiency (EQE; greater than 10% is desirable) [2,18]. The most popular are highly π-conjugated arenes (e.g., derivatives of carbazole, fluorene, terfluorene, pyrene, etc.) that have to be anchored properly onto the POSS core via chemical bonding.

The modification methods of silsesquioxanes are based on stoichiometric procedures, such as using prefunctionalized chlorosilane for hydrolytic condensation reaction or nucleophilic substitution. The catalytic transformations of POSS compounds are another synthetic approach and depend on the type and amount of a reactive group attached to the Si–O–Si core (e.g., Si–H or Si–HC=CH_2_, etc.). Certain types of transition metal (TM) catalyzed transformations have been applied to modify the abovementioned reactive groups through hydrosilylation (HS), cross-metathesis (CM) and coupling reactions (e.g., silylative coupling (SC), Heck coupling (HC), Sonogashira coupling, etc.). They may be used to attach a specific type of chromophore onto the rigid silsesquioxane core in order to obtain compounds of specific optoelectronic properties that can be applied in the formation of OLEDs devices.

The aim of this paper is to review the synthetic routes that lead to an efficient synthesis of silsesquioxanes with organic coronae of specific optoelectronic properties that constitute a group of electroluminescent (EL) and photoluminescent (PL) materials for the fabrication of electronic and optical devices. The crucial aspect is to designate a specific organic dye of a respective wavelength (color of emitted light) and anchor it to the POSS core using proper reaction procedure, which is dependent on the type of functional group at the Si–O–Si core.

## 2. Stoichiometric Reactions for the Synthesis of Functionalized Silsesquioxanes as Precursors of Photoactive Compounds 

This section concerns stoichiometric reactions leading to the synthesis of silsesquioxane-based systems for the fabrication of photoactive materials and their precursors. It should be noted that the most important class of stoichiometric processes concerns the hydrolytic condensation of respective, prefunctionalized chloro- and alkoxysilanes. This may be part of the greatest problem of the selectivity of obtained products, depending on the kind of solvent, pH, additives, concentration, time, and so on. However, at this stage, silsesquioxanes are obtained with functional groups (e.g., Si–H, Si–HC=CH_2_, Si–CH_2_CH_2_CH_2_X (X = NH_2_, Cl), etc.) and that is why it is so crucial. These POSS-based systems are used in further modification, and the form of the modifications is restricted to the nature of new functionality anchored onto the Si–O–Si core.

Hydrolytic condensation may be also used to obtain highly functionalized POSS-based compounds of interesting photophysical properties; however, the reports on this subject are rather rare; the reaction conditions may not be optimized sufficiently, and as a consequence result in rather low products yields. As an example, a paper by Lucenti et al. describes hydrolytic condensation (”corner capping”) of hepta(cyclohexyl)silsesquioxane trisilanol with respective tri(methoxy)silylpropyl pyrylene diimide to obtain pyrylene diimide with T_8_ unit, but only with a 23% yield [25]. This is described in Section 3.1, due to the ease of respective product comparison.

**Figure 2:**
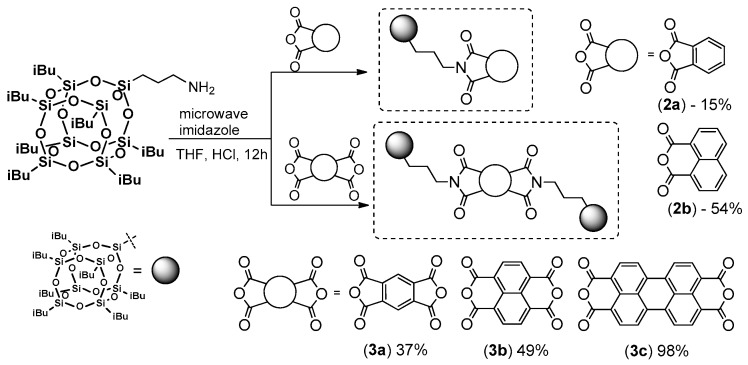
Synthetic path to obtain mono- and di-POSS-based imides and diimides.

The amidation or esterification processes between respective reactive groups are also worth mentioning. An interesting example of using (3-aminopropyl)hepta(isobutyl)silsesquioxane in the amidation of mono- and bis-anhydrides in microwave radiation was reported by Clarke et al. (Figure 2) [26]. However, irrespective of a fast reaction time, the reaction conditions, and especially the 4-fold excess of silsesquioxane that resulted in only (mostly) moderate to good yields of the respective products (**2a**,**b**, 15%–54%; **3a**–**c**, 37%–98%), which may not be satisfying. 

In their study, Clarke et al. present the photophysics of the compounds obtained. All products presented absorption bands (in solution, CHCl_3_) that were similar to and typical of their organic imide counterparts (λ_ab_ = 294 nm (**2a**), λ_ab_ = 309 nm (**2b**), λ_ab_ = 335, 350 nm (**3a**), λ_ab_ = 342, 360, 381 nm (**3b**), λ_ab_ = 459, 490, 526 nm (**3c**)). The emission spectra were much poorer for mono-POSS imide, as **2a** only had one wide band at ca. 330 nm, and for **2b** the strongest three peaks were at 362, 380 and 398 nm, respectively. However, the fluorescence quantum efficiency was very low for these two compounds, ca. Φ = 0.02. On the other hand, for the diimides, the resulting emission spectra were more accurate and reflected the presence of diimide unit (compared with organic counterparts), and three λ_em_ peaks were present (362–390 nm for **3a**, 350–404 nm for **3b** and 533–620 nm for **3c**). Only in the case of **3c** was the λ_em_ red-shifted with Φ that equaled 1.0. The low value of Φ suggests quenching of fluorescence due to an aggregation of organic moieties. In this case, only **3c** had potential for use in optoelectronic device fabrication.

**Figure 3:**
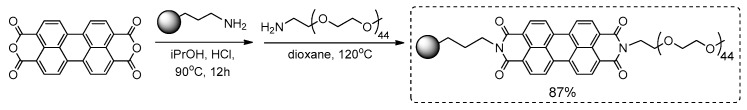
Reaction route for the synthesis of asymmetric pyrylene diimide.

A similar synthetic concept based on the amidation reaction of aminopropylPOSS and pyrylene anhydride, along with its consecutive amidation with methoxypolyethylene glycol amine, was presented by Bai et al. (Figure 3) [27]. However, the isolation yield of the intermediate product after the first amidation reaction with aminopropylPOSS reagents resulted in only a 23% yield, while the sequent amidation reaction that was conducted to obtain the final product—asymmetric pyrylene-based diimide—with 87% yield. As a result, the overall yield of the final product was 20%.

Nevertheless, this compound exhibited interesting photophysical properties. As reported, pyrylene diimides are strongly susceptible to concentration quenching due to intermolecular π–π stacking [28]. In POSS-based pyrylene diimides, the POSS unit prevents the aggregation of pyrylenes, resulting in decreased emission quenching, and these compounds exhibit a strong excimer-like red emission at ca. 620–660 nm (λ_ex_ = 495 nm) [26,29]. The compounds described above were tested as chemosensors for variety of anions (F^−^, Cl^−^, Br^−^, I^−^, NO^3−^, AcO^−^, ClO^4−^ and H_2_PO^4−^), but only in the case of fluorine anions was the red emission found to be quenched, along with its increasing concentration. Interestingly, this was a result of silsesquioxane cage hydrolysis catalyzed by F^−^, as previously reported in [30,31,32]. The decomposition of POSS units enables a consecutive and induced aggregation of pyrylene units and results in quenching the fluorescence phenomenon. This, in turn, offers a new optical strategy for toxic F^−^ ion sensing.

Bai et al. also presented a synthesis of asymmetric pyrylene-based diimine with thePOSS unit as pendant moiety and *N*-isopropylacrylamide unit that was subjected to atom transfer radical polymerization (ATRP), resulting in a well-defined amphiphilic fluorescent polymer with a 58% yield [29]. Their fluorescence red emission bands were retained at ca. 645 nm (λ_ex_ = 495 nm) and were found to be temperature-dependent (intensity enhanced with an increase in temperature). This could be used to fabricate thermo-responsive materials, in biosensors, and so on. 

**Figure 4:**
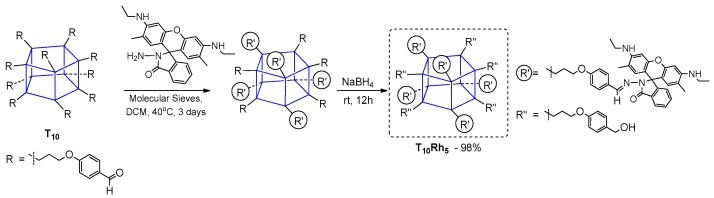
Synthetic path to obtain rhodamine B hydrazidedecorated T_10_ via an imidation reaction.

The imidation reaction was used by Ervithayasuporn et al. to anchor rhodamine B hydrazide units using propyloxy-p-benzaldehyde-functionalized T_10_ silsesquioxane (Figure 4) [33]. The precursor reagent T_10_ was obtained via nucleophilic substitution cage rearrangement of octa(chloropropyl)silsesquioxane, but reported reaction conditions that resulted in a 15% yield of T_10_.

Here, the authors reported on the synthesis of penta-substituted rhodamine B hydrazide T_10_ derivative with very high (98%) yield. The bulkiness of rhodamine B hydrazide moieties enabled the introduction of only five units onto the Si–O–Si core. Nevertheless, the photophysical properties of this compound were found to be interesting in the presence of Hg^2+^ ion, with absorption at λ_ab_ = 520 nm and red emission (λ_ex_ = 520 nm) that increased with the ion concentration rise. The observed active form that exhibited fluorescence was a spiro-lactam form of rhodamine B unit after Hg^2+^ chelation. Analogous tests were performed for others metal ions but none of them gave the same fluorescence response. This was a very interesting work enabling use of the presented compound as a selective dual chemosensor for Hg^2+^ that afforded both fluorescence enhancement and color change from colorless to pink, with a 0.63 ppb detection limit.

As reported by Tang, Xu and co-workers [34], an example of the parallel amidation and esterification of 4-(1,2,2-triphenylvinyl)benzoic acid used for the modification of octa(aminopropyl)POSS and octa [hydroxypropylodi(methyl)siloxy]POSS, respectively, resulted in the formation of analogous octafunctionalized silsesquioxanes differing from each other with the spacer (containing amide or ester functionality) between POSS and organic arene coronae. These two systems were obtained with 77% and 58% yields. Although these two compounds exhibited absorption spectra (in THF) at around λ_ab_ = 325 nm, the effective emission band was presented only for the amide derivative (a wide of spectrum ranging from 375 to 600 nm) with a maximum λ_em_ = 458 nm (λ_em_ = 365 nm). This compound was found to be a selective chemosensor for Cu^2+^ ions (other TMs were also tested) by fluorescence quenching in DMSO.

Anchoring the POSS unit on 3,4-ethylenedioxythiophenes was explored by Önal and Cihaner et al. [35,36,37]. They proposed an amidation reaction of amonipropyl(heptaisobutyl)POSS with anhydride, followed by a palladium-mediated Stille reaction to obtain the respective reagents, with moderate (44%–59%) yields. These systems were reagents in the electrochemical (co-)polymerization, resulting in highly conjugated poly(3,4-ethylenedioxythiophenes) (PEDOTs) known for their interesting electrochemical properties. PEDOTs can be used as elements in electrochromic device fabrication because their color depends on the oxidation state.

Rogach and Choy [38,39] reported the use of mono(3-mercaptopropyl)heptaisobutyloctasilsesquioxane as a dopant in the formation of solid stable perovskite (CsPbX_3_ (X = Br or I)) colloidal nanocrystals (NCs), with a surface protection of POSS as luminophores exhibiting different emissions colors. This compound may be obtained via the hydrolytic condensation of (3-mercaptopropyl)trimethoxysilane with trisilanol form of not-completely condensed silsesquioxane (iBu)_7_(Si_7_O_9_)(OH)_3_, but it is also commercially available. The synthetic procedure for obtaining of the final material consists of a simple formation of sulphides bonded (-S-metal) between the nanocrystals and POSS in toluene (added in different stoichiometry). Silsesquioxane, which acts as a hole-blocking layer, is optically transparent and also prevents the NCs from aggregation.

## 3. Catalytic Reactions Leading to Functionalized Silsesquioxanes as Building Blocks for Photoactive Materials

The synthetic chemistry of organosilicon compounds, including silsesquioxanes, has been profoundly dominated by the TM-mediated catalytic process. This is dictated by high effectiveness and selectivity (regio- and stereo-) of these catalytic transformations. In the case of silsesquioxanes, the proper choice of functional group, as well as the amount anchored to the Si–O–Si core, affects the selection of the respective catalytic reaction to be applied for the modification. As mentioned in Section 1, the silsesquioxanes with Si–H, Si–HC=CH_2_ or Si–aryl–X (X = I, Br, etc.) have been of the utmost importance due to their easy catalytic modification via hydrosilylation, cross-metathesis or coupling processes.

### 3.1. Heck Coupling for Obtaining Silylalkenyl Units Anchored at the Si–O–Si Core

Heck coupling (HC), also known as the Mizoroki–Heck reaction, is generally a palladium catalyzed coupling process between unsaturated C=C bonds (here, Si–HC=CH_2_) and an aryl halide (usually –I, –Br) resulting in the formation of alkenyl-substituted arenes. It occurs in the presence of additional phosphine ligand and equimolar amount of a base, although there are many variations of this process, a wide scope and tolerance to many organic functional groups is observed [40].

**Figure 5:**
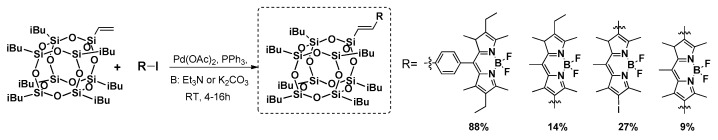
Respective structures for BODIPY derivatives of POSS.

Mono-functional silsesquioxanes are generally less frequently applied in obtaining compounds with interesting photophysical properties. Nevertheless, there are a few interesting examples to present that reflect the application of Heck coupling as a method for their synthesis. It is worth mentioning that the compounds are characterized by better thermo- and photostability. Liras et al. presented the synthesis of boron dipyrromethane (BODIPY) derivatives of POSS and their photophysical characterization (Figure 5) [41].

The elaborated synthetic procedure was not so effective, and enabled the respective mono- and disilsesquioxane substituted BODIPY derivatives (BODIPY–POSS) to be obtained with low (9%) to high yields (88%). Their photophysical properties were determined in EtOAc solution and poly(methyl methacrylate) (PMMA) film. Maxima absorption wavelengths were similar in both systems, with λ_ab_ in a range of 521 to 536 nm, and maxima fluorescence emission wavelengths λ_em_ ranging from 532 to 575 nm. Thermal stability was improved for both systems, i.e. mono- and disilsesquioxane substituted BODIPY derivatives. The most photostable hybrid dyes were the systems with a POSS unit linked to the BODIPY by one or two ethylene bonds (π-conjugation). The results obtained indicate a possibility for these systems to be applied where high thermo- and photostability are required.

Monovinylhepta(cyclohexyl)silsesquioxane was reported as an efficient reagent towards Heck coupling (mediated by Pd(P^t^Bu_3_)_2_]) with p-iodophenyl pyrylene diimide by Lucenti et al. [25]. This compound synthesized with a moderate 37% yield when compared with an analogous one, obtained via stoichiometric hydrolytic condensation (mediated by MeC_6_H_4_SO_3_H) of hepta(cyclohexyl)silsesquioxane trisilanol with respective tri(methoxy)silylpropyl pyrylene diimide (23% yield). These two systems, where POSS unit and pyrylene diimide moiety were separated either via an aliphatic (propyl) or styryl spacer, were analyzed in terms of their absorption/emission spectra and compared to their pyrylene diimide counterparts. The type of the linker (i.e., aliphatic or arene) did not bear any influence on the photophysics of these. The analyses as expected showed slight red-shift emissions of up to 10 nm in solution (CHCl_3_), but with very good photoluminescence quantum yields (up to 100%). The analogous analysis for solids resulted in higher red-shifted emission spectra due to the expected aggregation and π–π intermolecular interactions. However, the photoluminescence quantum yields for the POSS-based systems were twice as high as for the respected organic analogue that suggests suppressed aggregation. This favors its possible use in the fabrication of efficient emissive devices. This part is presented in this section for the ease of product comparison, despite the fact that it also concerns condensation reactions.

**Figure 6:**
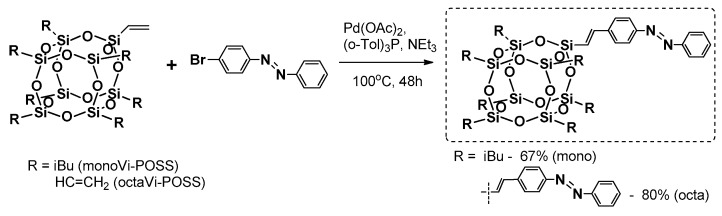
Mono- and octa-azobenzene-functionalized T_8_ derivatives obtained in a Heck coupling reaction.

Heck coupling was also applied by Liu et al. in the synthesis of thermally stable mono- and octa- azobenzene-functionalized T_8_-based dyes (Figure 6) [42]. The procedure enabled formation of the designed compound with yields of 67% and 80%; however, the reaction conditions were more severe in comparison with the previous example. Both systems were proved to be thermally stable with *T*_d_^5%^ = 273 and 383 °C for the mono and octa products, respectively. Introduction of these azobenzene moieties reduced the crystallinity of the compound withthe increasing amount of these groups in the molecule. The trans–cis photoisomerisation was examined and supported by DFT (Density Functional Theory) calculations. These systems were fluorescent with a maximum emission wavelength λ_em_ at about 400 nm, which enables their potential application as blue light emission materials.

Octa-functional POSS-based OLEDs have attracted far more attention mainly due to the possibility to anchor chromophores moieties (i.e., eight groups) onto the Si–O–Si core. Heck coupling may be conducted in a few combinations and towards the formation of fully substituted products as well as without full substitution.

**Figure 7:**
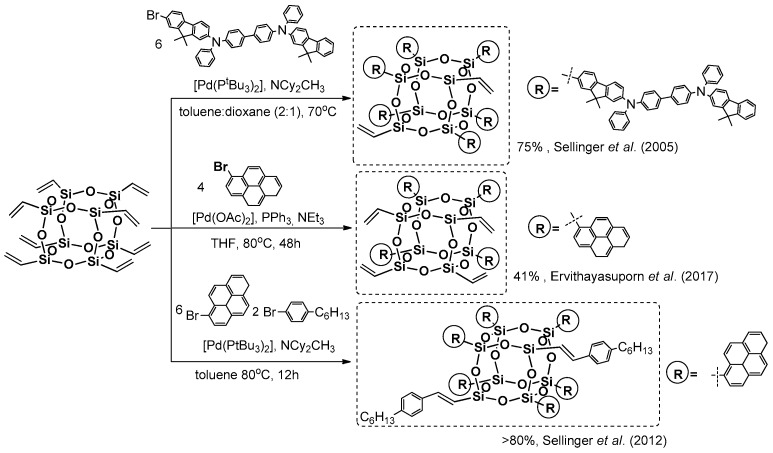
Heck coupling reaction leading to formation of T_8_-based systems with two functionalities.

Pioneering work was presented by Sellinger et al. when they reported on the use octavinyl silsesquioxane (OVS) in a Heck coupling process with a mono-brominated version of a highly π-conjugated arene system in the presence of [Pd(P^t^Bu_3_)_2_], obtaining hexa-substituted OVS with a 75% yield (Figure 7) [43]. Incomplete substitution was found probably due to a steric hindrance of the chromophore used and steric availability of vinyl groups. The obtained compound was verified in terms of its thermal stability, showing very high *T*_d_^5%^ = 465 °C. Its film and solution (toluene) photoluminescence spectra revealed λ_em_ = 430 and 434 nm respectively. The resulting electroluminescent device showed 18% improvement in external quantum efficiency over their small organic chromophore analogue.

Analogously, not-fully-substituted pyrene-based T_8_ cages were obtained by Ervithayasuporn et al. in Heck coupling using a [Pd(OAc)_2_]/PPh_3_/NEt_3_-mediated reaction that, after two days, resulted in the formation of a product with four pyrene substituents (as the most abundant species) with a 41% yield (Figure 7) [44]. The photophysical properties of the product were determined and broad emission bands λ_em_ = 406, 426 and 494 nm of low intensity in DMSO and sharp λ_em_ = 398, 421 and 445 nm of high intensity in THF were revealed. The compound revealed the solvent’s polarity dependant fluorescence, the π–π* emission of sole pyrene moieties was observed in THF, while in DMSO the formation of a pyrene–pyrene excimer through space has been postulated (the authors suggest its confirmation due to a large Stokes shift of Δλ = 143 nm). This compound was also tested as a sensor for several types of anions and, interestingly, was proved to encapsulate fluoride that results in a π–π* fluorescence enhancement in DMSO. Recent research by Ervithayasuporn et al. is related, with the synthesis of an analogous compound, but with four anthracene substituents (Figure 7) [45]. The synthetic procedure was based on Heck coupling reaction with the same catalytic system to obtain the desired product with 86% yield. Its photophysical parameters disclosed similar solvent’s polarity dependant fluorescence. It was also used to perform additional tests for possibility to encapsulate a variety of anions (e.g., F^−^, OH^−^, CN^−^ and PO_4_^3−^) by observation of fluorescence quenching due to from charge-transfer complex formation. This resulted in the possibility to use this compound as a sensor of the abovementioned anions.

Heck coupling to yield pyrene derivatives was also performed by Sellinger et al. They first reported on octavinyl T_8_ cage functionalization, performing a [Pd(P^t^Bu_3_)_2_]-mediated reaction with bromopyrene [46]. This was performed to obtain complete substitution (8–substituted as most abundant–P8) in comparison to the double Heck reaction product (14–substituted as most abundant–P14) with a >80% yield that was thermally stable up to 495 °C. These two systems were also analyzed in terms of their photoluminescence properties in solution (toluene), λ_em_ = 425 nm (P8) and 431 nm (P14), as well as in thin films, λ_em_ = 494 nm (P8) and 506 nm (P14). This meant a 70–75 nm red-shift effect when compared results in solution vs. in a solid state that suggested possible pyrene unit aggregation. The problem of two high “density” pyrene units was resolved by performing an efficient functionalization of vinyl-substituted T_8_, T_10_ and T_12_ cages with bromopyrene and a mixture of bromopyrene and a smaller steric reagent (i.e., 4-heptylbenzene) using the same catalytic Pd system (Figure 7) [47].

Obtained products (i.e., monopyrene(heptaisobutyl)silsesquioxane, along with the pyrene/heptylstyrene-substituted T_8_, T_10_ and T_12_ cage product mixture) were analyzed with TGA (ThermoGravimetric Analysis), showing their high thermal stability up to 359–469 °C. The films prepared (using solution-processed deposition) from the obtained materials exhibited a sky-blue emission (λ_em_ in the range of 459–486 nm for thin films, and λ_em_ in the range of 400–449 nm for the toluene solution). These results may also suggest chromophore aggregation in a solid state. The obtained compounds were tested in solution-processed OLEDs prepared to obtain materials showing current efficiencies of 3.64% and 9.56 cd A^−^^1^, respectively.

**Figure 8:**
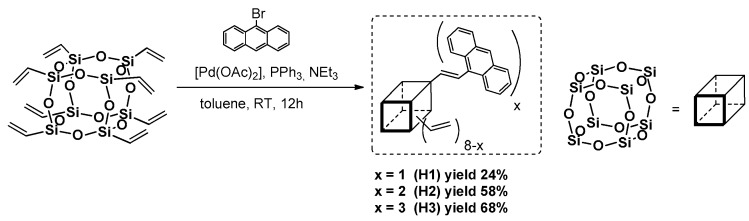
Heck coupling of OVS leading to mono-, di- and tri- anthracene-functional POSS.

Xu et al. presented a Heck coupling-based strategy to obtain anthracene-substituted T_8_ cage via a [Pd(OAc)_2_]/PPh_3_-catalyzed reaction at room temperature in various reagent stoichiometry resulting in differently substituted products (Figure 8) [48]. The substitution was also statistical, but with the majority of the presented forms of products that depended on the possibility to form a complex between palladium with bromoanthracene substrate.

Their *T*_d_^5%^ in the range of 407 °C (H3), 468 °C (H1) and 480 °C (H2), respectively, suggest high thermal stability in comparison with bromoantracene *T*_d_ = 246 °C. The photophysics were verified and the UV-vis spectra revealed the presence of three absorption maxima wavelengths λ_ab_ = 356, 373 and 390 nm for solution in toluene. The emission spectra were recorded in toluene, showing emission maxima wavelengths λ_em_ = 440 (H1), 461 (H3) and 458 (H2) nm, which were compared to the spectra for solids that disclosed red-shifts Δλ = 42–51 nm, suggesting known phenomena of possible anthracene unit aggregation (the smallest effect for H1). The same authors reported on the further modification of dianthracene(hexavinyl)octasilsesquioxane (H2), and using Heck coupling to introduce one diazophenylnaphtalenediamine moiety followed by an amidation process to introduce a complex structure of a chromophore that possess broad-band absorbing properties [49]. The presence of different chromophores allows the absorbing bands of the dyes to be combined, reduces the possibility of their aggregation and possesses a broad red-emission band at λ_em_ = 638 nm.

**Figure 9:**
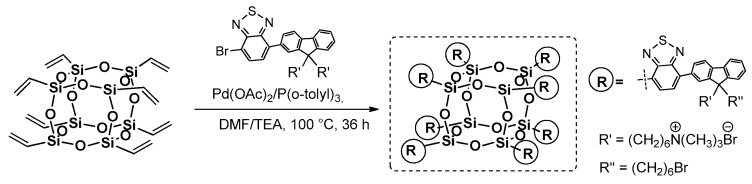
Efficient Heck coupling for the formation of octa-substituted T_8_ with conjugated fluorene and benzothiazole units.

Completely octa-substituted T_8_ cage obtained via Heck coupling reaction was reported by Liu et al. and used for the synthesis of a water-soluble hybrid nanodot based on POSS and conjugated electrolytes for the Two-Photon Excited Fluorescence (TPEF) analysis that imaginesthe cellular and cancer nuclei (Figure 9) [50,51].

The Two-Photon Excited Fluorescence is a microscopic method used to reduce photodamage to a cell, improve resolution and minimize cellular autofluorescence, but it has rarely been exploited, due to the lack of efficient absorption materials that can enter nuclei and in turn selectively stain them. The desired fluorophore was anchored to the POSS core via a Heck coupling procedure of octavinyl silsesquioxane (OVS) and respective monobromo-substituted chromophore in the presence of [Pd(OAc)_2_/P(*o*-Tol)_3_] with a 45% yield. The quaternized silsesquioxane derivative was soluble in water and its average diameter of 3.6 ± 0.3 nm enabled easy penetration of cellular nuclei. The photophysical properties were verified and absorption maxima λ_ab_ bands at 320 and 465 nm corresponded to the fluorene and benzothiazole units, but they red-shifted by 34 and 75 nm, respectively, due to the –HC=CH–POSS moiety extending the conjugation system. On the other hand, the λ_em_ was located at 617 nm and exhibited a large Stokes effect at 152 nm.

**Figure 10:**
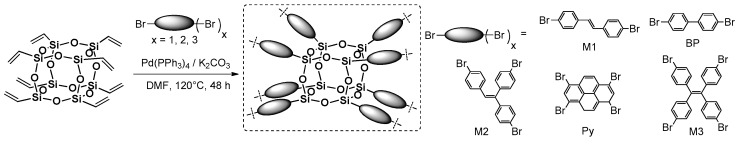
Synthetic route leading to hyperbranched 3D POSS-based materials obtained via Heck coupling.

An interesting scientific concept was proposed and performed by Liu et al., who used octavinyl silsesquioxane (OVS) and multibromo-substituted arenes in a Heck Coupling process to yield crosslinked 3D networks of highly hyperbranched structures with a >80% yield (Figure 10) [52,53].

The reaction procedure was also modified by using mixtures of different chromophores (e.g., BP and Py) in various stoichiometry, which resulted in the formation of macromolecular 3D systems with different chromophore content. The purification procedures of obtained polymeric materials, due to their 3D structure, was necessary due to the presence of non-reacted comonomers, and post-reaction inorganic salts and other residues within the matrix. The isolation was mostly based on filtration and washing with different solvents. For this reason, a Soxhlet apparatus was used with THF and MeOH to obtain pure products. All of the obtained compounds were analyzed in terms of their proven thermal resistance and high *T*_d_^5%^ at ~390 °C, but the high porosity of these materials was revealed by during BET (Brunauer–Emmett–Teller) analysis. Depending on the type (and amount, if mixture) of respective arene, respective hyperbranched products exhibited various surface areas from 358 to even 848 m^2^g^−1^ (when M3 was used as a reagent). The most interesting part of the research was disclosed in defining the photophysical properties of both substrates (M1–M3, BP, Py). In the case of M1, M2 and M3, which showed high blue fluorescence (λ_em_ at ca. 435 nm) in solid state and in solution (DCM = dichloromethane), the respectively obtained hyperbranched products showed strong red shifts (observed at λ_ex_ = 365 nm), and green (λ_em_ = 509 nm for prod.1, derived from M1; λ_em_ = 518 nm for prod.2, derived from M2) or yellow (λ_em_ = 540 nm for prod.3, derived from M3) fluorescence. This may be derived due to the extended π–π conjugation in the 3D network by additional –HC=CH– linkage. The ethanol dispersion of these materials was applied to obtain sensors for various nitroaromatic explosives by fluorescence quenching [53]. On the other hand, modulation of the stoichiometry of BP and Py in Heck coupling with OVS resulted in the formation of nine types of materials that altered the amount of BP and Py units in the frameworks. As a result, they exhibited continuous color change from bluish-green at λ_em_ = 485 nm, to green at λ_em_ = 528 nm, yellow at λ_em_ = 555 nm and finally to red at λ_em_ = 605 nm. High fluorescence quantum yields in the solid state were calculated and ranged from 2.46% to 22.42% [52]. These systems were used along with the thiol-containing polysiloxane matrix to produce a UV-LED device that shows color-transformable properties by simply turning the irradiation on and off.

**Figure 11:**
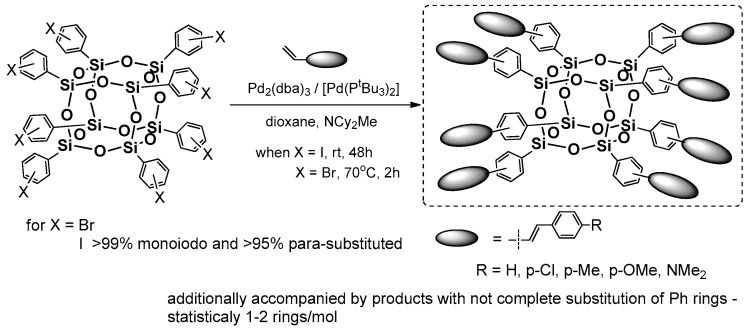
Heck coupling using halogenophenylPOSS reagents.

Heck coupling using the silsesquioxanes may be performed also in the reverse functional group placement (i.e., to applied octa(halogenophenyl)silsesquioxane and styrene derivatives as a source of –HC=CH_2_ moiety). Respective research on this issue was pioneered by Laine et al. [54]. The primary reagent for the study was cubic octa(phenyl)silsesquioxane (OPS) that was subjected to bromination via a Fe-catalyzed reaction that resulted in [octa(polybromophenyl)]silsesquioxane. The resultant compounds had a different substitution of bromine at the Ph ring, but this could be controlled by reaction stoichiometry to obtain a para-brominated 2,5-dibrominated derivatives. A more-efficient and selective procedure for the synthesis of analogously octa(p-iodophenyl)silsesquioxanes using ICl was also reported [55,56]. These two systems are perfect halogenophenylPOSS reagents for the Heck and other coupling processes (e.g., Suzuki-type) [57]. In 2010, Laine et al. reported on synthesis of a series of octa(stilbene)silsesquioxane derivatives using octa(p-iodophenyl)silsesquioxane and octa(bromophenyl)silsesquioxanes (on average 67% p-substituted, 24% meta, 9% ortho and 3% disubstituted) with respective p-substituted styrenes in Heck coupling reaction conditions (i.e., [Pd_2_(dba)_3_] (1 mol %)/[Pd(P^t^Bu_3_)_2_] (2 mol %) that resulted in octa(stilbene)silsesquioxanes with diverse p-phenyl substituents with high 62–86% yields (Figure 11) [58].

The desired octa-substituted products were also accompanied by compounds with not-completely substituted phenyl rings, which was a result of not-fully halogenated octa(phenyl)silsesquioxane, especially in the case of Br [54]. The corresponding products were characterized in terms of their thermal resistance (*T*_d_^5%^ in the range of 318–429 °C). The greatest emphasis was placed on their photophysics assessment and its comparison with trans-stilbene and Me-stilbene-Si(OEt)_3_. The UV-vis spectra of products (in THF) were similar in shape and red-shifted 5–10 nm (λ_ab_ 298–320 nm) to their molecular counterparts, but their large red shift of 60–100 nm was visible for the emission spectra of these systems when compared with trans-stilbene and Me-stilbene-Si(OEt)_3_ (λ_em_ 334–436 nm)_._ The greatest difference was noted for stilbene and p-Me-stilbene silsesquioxane derivatives that were also collated with UV-vis and PL spectra for octa(p-Me-stilbene)silsesquioxane and its hepta(p-Me-stilbene)phenylsilsesquioxane. Altogether, the absorption–emission changes that were observed along with the theoretical calculations and additional analysis of stilbene–siloxane and cyclosiloxane, respectively, suggest that the large red-shift may have resulted from interactions of the stilbene π* orbitals with a LUMO located inside the cage and involving all Si and O atoms, as well as the organic substituents that interact in the excited state. Interestingly, for the NMe_2_ derivative the solvatochromism phenomenon was observed mainly due to the charge–transfer interaction when the polarity of solvent was changed.

Studies on stilbene derivatives of silsesquioxanes were also conducted. One of the aspects to be studied was the usage of octa(halogenephenyl)silsesquioxane reagents substituted with bromine at phenyl ring in different places (i.e., octa(o-bromophenyl)silsesquioxane, but also octa(2,5-dibromophenyl)silsesquioxane and octa(2,4,5-dibromophenyl)silsesquioxane) [59]. This provided more functionalities per unit volume, exceeding the density when compared to POSS-based dendrimers. These compounds were used for analogous Heck coupling procedure (including catalytic systems, etc.), leading to corresponding octaphenyl silsesquioxane (OPS)-based styrenes with 8, 16 and 24 styryl substituents, with R = 4-Me, R = 4-NBoc and R = 4-Ac groups, respectively [60]. Interestingly, the thermal parameter for these systems is similar to their octa-substituted derivatives. The blue shift of the absorption and the red shift of the emission spectra for the (R-o-styryl)_8_OPS suggest the interactions of the Si–O–Si cage with the ortho-substituted organic groups. On the other hand, the (Rstyryl)_16_OPS exhibited a longer conjugation length, resulting in higher values of Stoke’s shift and (Rstyryl)_24_OPS behavior that revealed the existence of both a regular π–π* transition and a charge transfer, which could be explained by two excited states involving the LUMO inside the Si–O–Si cage.

**Figure 12:**
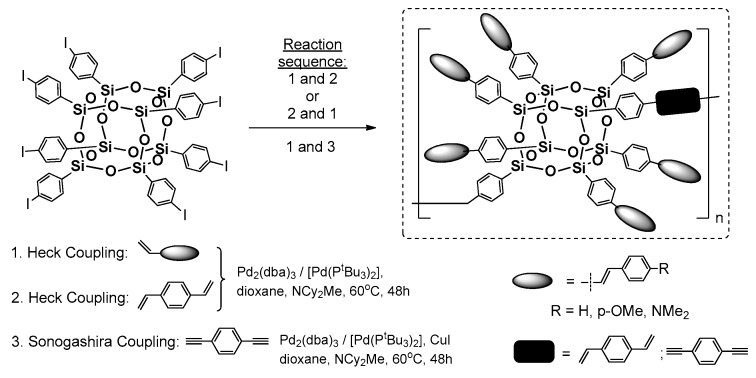
Synthetic approach for linear copolymers obtained via consecutive Heck and Sonogashira coupling.

The synthetic procedure of Heck coupling was also conducted in a controlled copolymerization of octa(p-iodophenyl)silsesequioxane with 1,4-divinylbenzene (DVB) and 1,4-diethynylbenzene (DEB). Laine et al. reported on a specific formation of copolymeric systems derived from octa(p-iodophenyl)silsesequioxane embedded between DVB or DEB fragments (Figure 12) [61].

The synthetic routes that were tested were based on changing the reaction sequence of octa(p-iodophenyl)silsesequioxane Heck Coupling functionalization (carried out with specific stoichiometry) and linking each silsesquioxane via Heck or Sonogashira cross-coupling with DVB or DEB. The procedure resulted in final copolymeric systems with a moderate 39%–51% yield and respected average molecular weights in the range of 9–30 kDa, as well as suggesting that the formation of di(p-iodostyryl)hexastyrylsilsesquioxanes as the first reaction sequence results in higher molecular weights of the resulting copolymeric systems. The photophysics of the two types of copolymers significantly depended on the organic linker. The DVB copolymers exhibited very little conjugation due to high 1,4-DVB fragments, whereas the DEB showed a strong red-shift at ~40 nm emission. This suggests electron delocalization through the Si–O–Si cage. Again, the p-aminostilbene copolymer derivative showed charge-transfer stabilization.

**Figure 13:**
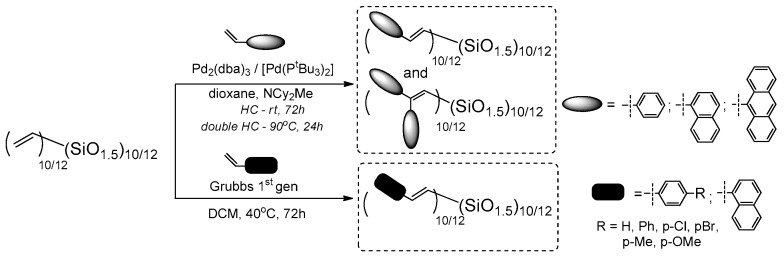
T_10_ and T_12_ arene derivatives obtained by Heck Coupling and Cross-Metathesis.

Laine and co-workers expanded the studies conducted on T_10_ and T_12_ cages with reactive Si–HC=CH, p-iodophenyl and bromophenyl substituents. They previously reported on the possibility of fluoride ion (TBAF = tetra-n-butylammonium fluoride) catalyzed cage rearrangements between polyvinyl- and polyphenylsilsesquioxane, resulting T_10_ and T_12_ with mixed reactive group placement (depending on the reaction condition and stoichometry) [30,31]. The research concerned using vinylT_10/12_ cage mixture and its functionalization using Heck and double Heck coupling with bromostyrene, 1-bromonaphthalene and 9-bromoanthracene, as well as Grubbs first generation catalyzed cross-metathesis of the vinylT_10/12_ with p-substituted styrenes (Figure 13) [62].

**Figure 14:**
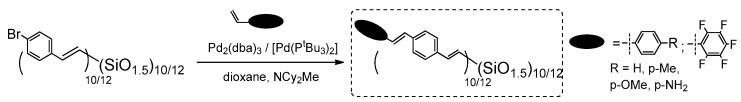
T_10_ and T_12_ stilbene derivatives obtained by Heck coupling and cross-metathesis.

In parallel, the reverse Heck Coupling (i.e., reaction) of (Br-styrenyl)T_10/12_ cage mixture with p-substituted styrenes was conducted (Figure 14).

The results for both processes are presented here for the ease of their comparison (CM is described in Section 3.3). The described catalytic processes are parallel routes for analogical T_10_ and T_12_ structure formations, and also similarly efficient. The products were compared, their thermal resistance was verified and they exhibited *T*_d_^5%^ up to 445 °C. The UV-vis and PL spectra in THF were analyzed and, in general, were similar to those obtained for T_8_ analogues. Interestingly, the UV-vis absorption maxima of products obtained via Heck and double Heck coupling were almost the same (in the range 254–374 nm), while the emission of double-functionalized compounds was a bit red-shifted (310–465 nm), as should be expected for the slightly extended, excited state of conjugation. The p-substituted stilbene-based T_10_ and T_12_ exhibited emission maxima dependent on the electronic properties on the substituents (e.g., the largest red shift of λ_em_ for the = –NH_2_ group, that results from a charge transfer). For this derivative, a solvatochromism was also observed for MeCN (acetonitrile), THF and cyclohexane solvents used. Additionally, the dependence of emission spectra shapes (maxima) and their intensity on the solvent used (MeCN:THF, hexane:THF) and the spectra collected for various compound concentration suggested their aggregation. This possible aggregation may be responsible for the exciplex formation and seems to differ significantly from the one known for traditional organic molecules. These findings might suggest smaller band gaps, meaning a possible use of these systems in 3D hole/electron transport devices.

The research connected with the use of T_10_ and T_12_ cages was carried on for copolymeric systems. Again, Laine et al. performed experiments using octa(vinyl)silsesquioxane T_8_ (OVS) and octa(p-iodopheyl)silsesquioxane T_8_ to obtain a mixture of T_10_ and T_12_ with mixed vinyl- and p-iodophenyl-substituent placement (with a 90% yield) along with an analogous reaction for vinyl and phenyl-substituted T_10_ and T_12_ cages [63]. The vinyl/phenyl-mixed T_10_ and T_12_ systems were substrates for the following:

- Heck Coupling reaction (analogous catalytic conditions described in [61]) with 1,4-dibromobenzene and 4,4′-dibromostilbene, resulting in lightly branched non-linear copolymeric systems with a 83–86% yield. This is the fastest way to obtain the desired copolymeric compounds.Cross-metathesis (described in detail in Section 3.3) reaction, using Grubbs first generation catalyst with 1,4-dibromobenzene to obtain (again) slightly branched non-linear copolymeric system with a 89% yield.The vinyl/(p-iodo)phenyl-mixed T_10_ and T_12_ cages were substrates for the reverse synthetic path (i.e., cross-metathesis reaction, using Grubbs first generation catalyst with 1,4-dibromobenzene to obtain a slightly branched non-linear copolymeric system with a 95% yield).The abovementioned copolymer (i.e., its p-iodophenyl substituents) was post-modified via a Heck coupling reaction using R-styrenes (H-styrene, OMe-styrene) to obtain R-styrene functionalized copolymer with a 67% yield.

All these six types of copolymers were tested in terms of their photophysical properties, with results collated for those with bis(vinyltriethoxysilyl)benzene and 4,4′-bis(vinyltriethoxysilyl)stilbene used as models. All copolymers exhibited a red shifted emission spectra that were strongest for the copolymers obtained via Heck Coupling (described in the first point) at ca. 20–60 nm for the DVB derivative when compared with the model compounds. This is attributed to shorter conjugated linkers, enabling Si–O–Si cage participation in the 3D excited states. The compounds were obtained in particular because of their 3D interaction in the excited state that corresponds with their application in OLEDs devices. 

### 3.2. Sonogashira Coupling for the Introduction of Silylalkynyl Moieties onto the Silsesquioxane Core

Sonogashira coupling (also known as Sonogashira–Hagihara) is a palladium-mediated reaction between an unsaturated –C≡CH terminal alkyne moiety and an aryl halide (usually –Br, –I, –OTf) that results in the formation of substituted alkynes. It is conducted in the presence of copper halide and requires stoichiometric amount as a base. The variations of this process are numerous and concern catalytic systems (including additional ligands), but it is a useful process in organic and organosilicon chemistry, and tolerant of a number of functional groups [64].

**Figure 15:**
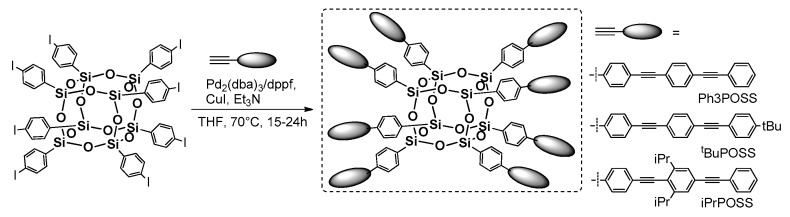
Synthesis route for octa-substituted T_8_ phenylacetylenes obtained via Sonogashira reaction.

This type of coupling requires the presence of –C≡CH or –phenyl-halogen moiety attached to POSS core. The first reports on this process in silsesquioxane chemistry concerned using octa(bromophenyl)POSS and octa(iodophenyl)POSS derivatives with phenylacetylnes in [Pd_2_(dba)_3_] and [Pd(PtBu_3_)_2_] catalyzed reaction, conducted at RT and 60 °C for 48 or 24 h, respectively and resulting in 56%–90% yield for –Br product, and 67%–90% yield for –I derivative [65]. In 2016, Chujo et al. applied this procedure using octa(iodophenyl)POSS with π-conjugated phenylacetylenes (Figure 15) [66]. These compounds however, were obtained with rather moderate yields (i.e., Ph3POSS = 20%; tBuPOSS = 19%; and iPrPOSS = 22%).

Nonetheless, all of these systems were verified in terms of their thermal resistance which was proved to be high (*T*_d_ = 496–526 °C). Their photophysical properties were disclosed via UV-vis and photoluminescence analysis and compared with their organic, trimthylsilyl analogues. The absorption spectra revealed the presence of two maxima wavelengths in the area λ_ab_ = 323–327 and 347–350 nm, and did not present any significant changes regarding the structural differences of substituents. The emission spectra were recorded in solution (CHCl_3_) and in a solid state. The relatively broader and red-shifted emission was recorded for Ph3POSS along with higher quantum efficiency in comparison to the organic analogue. This was explained by the inorganic core presence as well as the mobility and π-conjugation of phenyl rings (undisturbed for alkyl derivatives). On the other hand, a strong, red-shifted λ_em_ maxima at ca. 405–427 nm was recorded, resulting from unfavorable intermolecular interactions. The authors reported high thermal resistance for the luminescence properties (after UV irradiation λ = 365 nm) that were maintained even at 250 °C.

### 3.3. Cross-Metathesis and Related Modfications for Silylalkenyl Fragment Formations

Cross-Metathesis (CM) is one of the fundamental catalytic transformations in the chemistry of organosilicon compounds [67]. In this manner, it is a TM-carbene-mediated (TM = Ru, Mo, etc.) process of C=C bond cleavage and formation of unsaturated organosilicon derivatives. Silsesquioxanes, which are promising precursors with Si–HC=CH_2_ reactive moiety may be placed in this group of reagents [68,69,70,71]. CM may be applied as a single synthetic route leading to desired POSS-based compounds, or used as a valuable tool for obtaining precursors of silsesquioxanes with reactive groups for consecutive reactions (e.g., Heck or Sonogashira coupling). In this matter, the described process was used to obtain a series of compounds exhibiting interesting photophysical properties.

**Figure 16:**
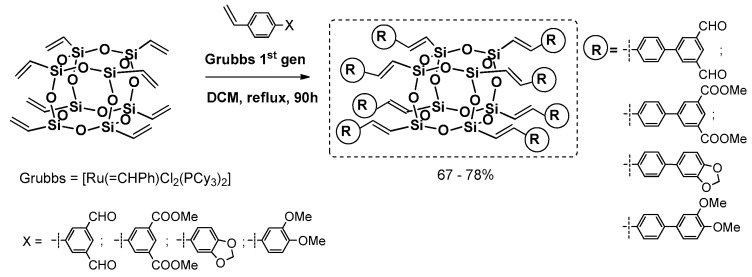
Cross-metathetic reaction path for the synthesis of octa(styryl)silsesquioxane.

Cole-Hamilton et al. contributed to this issue with the use of octavinyl silsesquioxane (OVS) grafted with styrene derivatives using Grubbs first generation catalyst (4 mol %) and 2–3 equiv. of styrene per one Si–HC=CH_2_ group and resulting in good 67%–78% product yields (Figure 16) [72,73]. The modifications of styryl rings resulted in changes in absorption and emission spectra, especially when aldehyde moiety was introduced and resulted in quenching emission. The POSS derivatives were reported to exhibit red shifts and broader spectral emissions due to the involvement of a silsesquioxane core in electronic delocalisation. It should be noted that CM reaction conditions may be improved so that not as much styrene excess is required to complete OVS conversion (24 h) [74].

**Figure 17:**
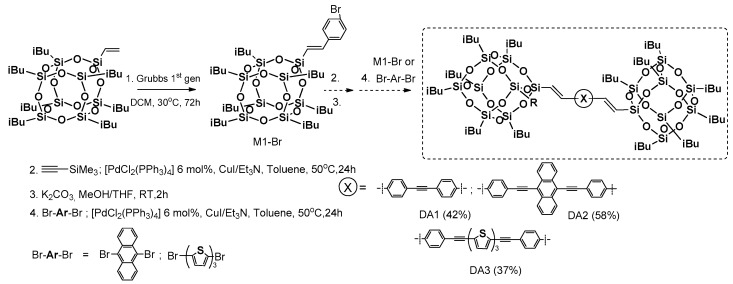
Synthetic reaction route based on sequential cross-metathesis and Sonogashira coupling for dumbbell-shaped π-conjugated systems with POSS as pendant groups.

Cross-metathesis was employed as precursor synthetic process for obtaining mono(p-bromostyryl)heptaisobutylsilsesquioxane (M1-Br) by Naka et al. [75]. This compound was applied as a reagent in two subsequent Sonogashira coupling reactions, resulting in dumbbell-shaped π-conjugated systems with POSS as pendant groups (Figure 17).

Unfortunately, the reaction conditions were based on the use of 3 equiv. of para-bromo-styrene, and 3 mol % of Grubbs first generation catalyst (added to the reaction in two portions) enabled the formation of precursor M1-Br with only a 68% yield, contrary to the procedure developed in our group [76]. The next steps of the process were performed with moderate 37%–58% yields of respective final dumbbell products (DA13). These were characterized in terms of their photophysical and thermal properties in comparison with their organic counterparts. Their *T*_d_^1%^ was respectively high (268–282 °C). The interesting part of the research concerns absorption and emission spectra performed in a solution vs. in a solid. The λ_em_ maxima in the solid state were red-sifted in a range of 34–50 nm and equaled 401, 542 and 520 nm for DA13, respectively. This was noticed after UV irradiation (352 nm) that revealed blue, yellow and greenish-yellow emissions in POSS-based systems contrary to their organic analogues. These results also indicate the participation of the Si–O–Si cage in the delocalization of electrons, and prevention of the π-conjugated parts from interaction. 

**Figure 18:**
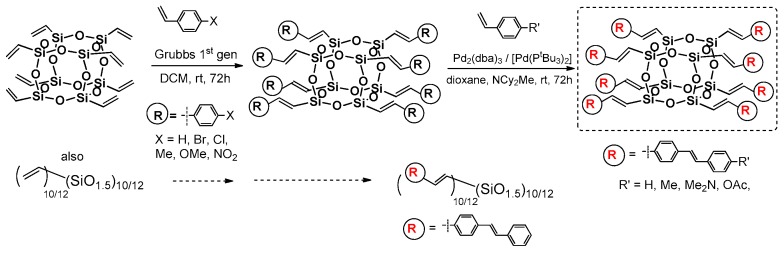
Reaction path for the selective sequence of cross-metathesis and Heck coupling towards (E)-styrenyl-substituted T_8_, T_10_ and T_12_.

Laine et al. have profoundly contributed to the development of cross-metathetic transformation using the most useful reagent in this process (i.e., octa(vinyl)silsesquioxane T_8_ (OVS), but also decavinylT_10_ and dodecavinylT_12_ cages). The selective introduction of substituted (E)-styrenyl moiety at Si–O–Si cores and for the bromo-derivatives enabled their consecutive modification via Heck coupling (Figure 18) [71,77]. Interestingly, the cross-metathesis for OVS proceeded with 0.5 mol % of the catalyst and at room temperature only in the cases of T_10_ and T_12_ at 40 °C. The purification of these compounds concerned filtration over Celite, precipitation and column chromatography, obtaining final products with 74%–82%yields. Additionally, the p-NH_2_-vinylStilbene product was transformed into respective benzamide derivatives. All of the compounds were characterized in terms of photophysical properties. For T_8_ compounds, the absorption (λ_ab_ 260–358 nm) and emission (λ_em_ 304–482 nm) spectra showed red shifts in comparison with their organic counterparts (styrene and p-vinylstilbene).

The octa(XStyryl)silsesquioxane T_8_ showed smaller red-shifts than octa(R′Stilbenevinyl) silsesquioxane T_8_compounds (10–40 nm shifts vs. 30–80 nm shifts—i.e., Stokes shifts at ca. 50 nm) as might be expected due to a larger conjugation for the R′StilbenevinylT_8_ compounds. The red shift is also larger in the presence of more electron-donating groups (–OMe). The NH_2_StilbeneVinylT_8_ exhibited effects of solvent polarity on its emission behavior (DCM and MeCN) due to charge-transfer (CT) interactions that were also proved by DFT calculations, as noted previously [58]. The StilbeneVinylT_10_ and T_12_ derivatives were separated effectively due to the proper solvent choice. Their photophysical properties were compared along with the StilbeneVinylT_8_ to observe the impact of the cage size and symmetry measured in solution (THF) and films. In general, the red-shift emission was observed for all three compounds, due to the π-conjugation, and the additional red-shifted spectra for films revealed the aggregation of chromophores, as expected. Additionally, two-photon spectroscopy analysis was performed to compare the polarization and non-linear absorption properties of the separated T_8_, T_10_ and T_12_ cages along with the p-triethoxylsilylvinylstilbene and p-vinylstilbene. Due to the differences in symmetry, especially between T_10_ and T_12_ cages, these compounds displayed changes in the dipole moment of excitation and exhibited different emission maxima wavelengths, with excitation at λ_ex_ = 333 nm (T_10_ − λ_em_ = 386 nm, high intensity), λ_ex_ = 400 nm (T_10_ − λ_em_ = 453 nm) and λ_ex_ = 800 nm (T_10_ − λ_em_ = 450 nm, the highest intensity when compared with T_8_ and T_12_). Indeed, the observed impact on fluorescence decreased for bigger T_12_ cages, which may have been derived from the cage symmetry and/or the chromophores’ proximity. Moreover, a red-edge effect in these 3D molecular structures was observed (i.e., the red-shifted emission wavelengths upon shifting of the excitation wavelength to the red end of the abs. spectra. The highest effect was observed in the case of deca(Stilbene)silsesquioxane T_10_, which suggests a possible application of these compounds as a new class of hybrid materials offering switchable emissions.

The cross-metathesis process, leading to polymeric POSS systems (e.g., ADMET reaction), was described in Section 3.1 due to the ease of comparison of the products, and is analogous in structure to a Heck coupling reaction [63].

**Figure 19:**
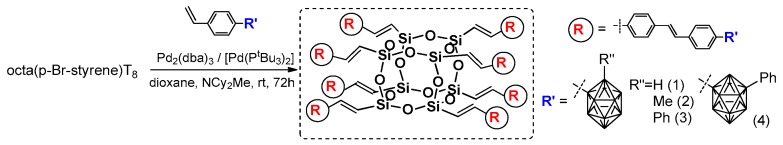
Synthesis of T_8_-based ortho- and metha-carboranyl-substituted vinylstilbenes.

The Núñez research group presented an interesting combination of analogous cross-metathesis and Heck coupling sequences leading to octa(carborane-Stilbenevinyl)silsesquioxane T_8_ cages (Figure 19) [78]. The cross-metathesis of OVS with p-bromostyrene was perfumed using the synthetic procedure reported by Laine [71]. As a result, octa(Br-styrene)T_8_ was applied as a reagent in efficient Heck coupling with four types of carboranyl-substituted vinylstyrene, producing a good isolation yield of 1–4 products (43–65%). The importance of this research lies in the combination of carboranes known from their 3D electron delocalization and the fact that they were additionally coupled with π-conjugated arene fragments along with the T_8_ silsesquioxane unit, enhancing thermal stability and also affecting the electronic properties of these systems. All compounds were characterized in terms of their absorption–emission properties (analysis performed in DCM) and supported by DFT calculations. The absorption spectra shapes were similar with λ_ab_ near 338 nm and all emission maxima wavelengths red-shifted to ~392 nm (λ_ex_ = 340 nm). Interestingly, the highest symmetry of product 1 in comparison with products 2–4 (Figure 19) was visible in the broadening of fluorescence in the red part of the spectra that indicates intermolecular interactions of the o- and m-substituted carborane units. This also caused the fluorescence quantum yield to be the highest for 1 (~60%). Additionally, the emission spectra of drop-casted films revealed its strong bathochromic effect (λ_em_ 450–484 nm) due to the aggregation and intermolecular interactions of substituents. However, these systems exhibited remarkable thermal resistance, with up to 87% residue on the initial weight at 1000 °C, making them excellent candidates for luminescent materials.

### 3.4. Metallative Coupling (Si, Ge) as a Complemenatry Catalytic Reaction towards the Formation of Alkenylsilyl Moieties

The importance of catalytic transformations in the chemistry of organosilicon compounds is undeniable, and the reactivity of the transition metal–p block element (TM–E) bond often determines the kinetics and selectivity (regio- and stereo-) of these processes. This aspect also concerns silsesquioxanes. A class of catalytic processes attractive in the organosilicon chemistry is silylative coupling (trans-silylation), discovered and developed by the Marciniec group over 20 years ago. This is a process between vinylsilanes and olefins, involving the activation of the =C–H bond at the *α* and *β* carbon atom of the vinyl group and the C*_vinyl_*–Si bond in the vinylsilane molecule, with simultaneous elimination of the ethylene molecule catalyzed by TM–H/Si (TM = Ru, Ir, Rh, etc.) complexes (the insertion–elimination mechanism that we proved) [79,80,81,82]. This leads to unsaturated *E/Z* and *gem*-alkenylsilanes. Because the selectivity of this reaction is strictly controlled, it results in a selective formation of *E*-isomers depending on the reaction conditions and catalytic system. The silylative coupling may be considered a complementary process to the abovementioned cross-metathesis. Despite the fact that these two reactions proceed via different mechanisms and catalytic systems, they result (when optimized) in the selective formation of *E*-1,2-substituted silylalkenes (in contrast to silylative coupling, which may be regioselective towards isomeric 1,1-substituted alkenes).

**Figure 20:**
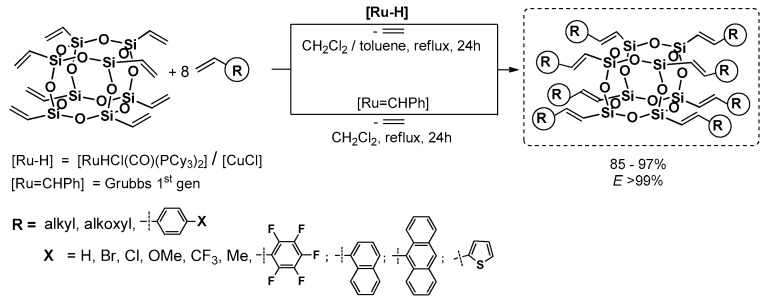
The silylative coupling vs. cross-metathesis leading to octa[(*E*)alkenyl]-substituted silsesquioxane T_8_.

In the chemistry of vinyl-substituted silsesquioxane modifications using both reactions, silylative coupling, metathesis and also hydrosilylation, our team gained a vast experience. In the scope of this research, it was shown that the studied processes could be used for the synthesis of unsaturated silsesquioxane-based systems using mono- and octavinyl silsesquioxanes (T_8_) as well as divinyl-substituted DDSQ reagents. The silylative coupling reaction mediated by the [RuHCl(CO)(PCy_3_)_2_]/[CuCl] system was found to be regioselective process leading to both mono- and octa[(*E*)alkenyl]-substituted silsesquioxane T_8_ cages (Figure 20) [74,76], as well as containing metalloids (Ge) [83]. The reactions were conducted with strictly controlled stoichiometry and were clean, as the only byproduct was the evolution of ethylene.

**Figure 21:**
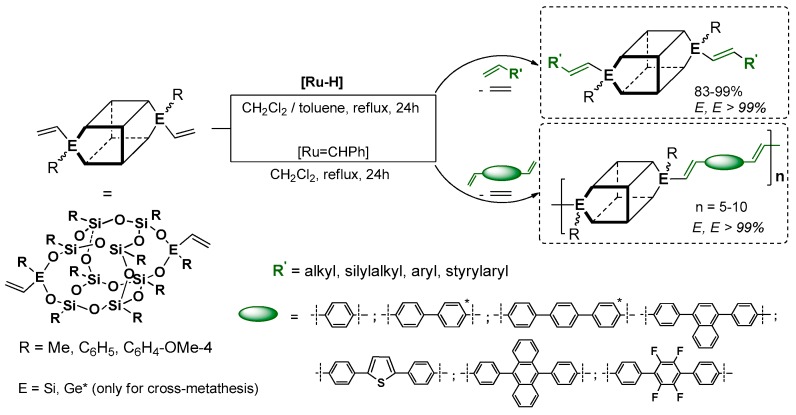
Synthetic protocol for stereoselective silylative coupling and cross-metathesis with olefins/dienes of divinyl-substituted DDSQ systems.

These research projects were expanded to a new member of a silsesquioxane family (i.e., a divinyl-substituted double-decker silsesquioxanes which were used as reactants in the silylative coupling and cross-metathesis (only for E = Ge) processes with a wide range of olefins, including highly π-conjugated arenes (Figure 21) [70,84,85,86]. It resulted in the development of a stereoselective method for the preparation of new molecular, dialkenyl-substituted DDSQ derivatives with a well-defined and documented structure ((*E*) isomer of –HC=CH–). The applied processes are a complementary tool for the synthesis of this type of systems unique in literature.

Interestingly, when dienes were applied, they rendered it possible to exploit the methodology for effective and stereoselective formation of new, macromolecular systems with the DDSQ core embedded in the copolymer chain and the preservation of silylene–ethylene–arylene fragments with double-bond (*E*) geometry. This catalytic protocole was the first example in the literature of the synthesis of macromolecular hybrid systems obtained via silylative coupling and cross-metathesis protocols in which the arene unit was stereoselectively connected by the ethenyl bridge with the DDSQ silsesquioxyl fragment.

This fragment concerns the silylative (metallative) coupling process which leads to the highly efficient and stereoselective formation of (*E*)-alkenylsilyl moieties that are anchored to the silsesquioxane core, irrespective of its structure (cubic or double-decker). It also concerns the amount of Si–HC=CH_2_ reactive groups (one, two or eight). Moreover, it may lead to the formation of both molecular and macromolecular silsesquioxane-based compounds. Due to the presence of the (*E*)-alkenylsilyl units, the resulting functionalized silsesquioxanes are complementary to the architecture of those compounds obtained via cross-metathesis and Heck coupling. Our methodology is an effective, straightforward and clean (only ethylene is a gaseous byproduct) route leading to these systems that are obtained from commercially available reagents. Due to the presence of the (*E*)-alkenylsilyl fragment and its conjugation with π-arenes, these molecules are potentially of high photophysical interest, as was proved in the case of the analogous POSS-based systems described previously in this review in chapter 3.1 and 3.3. As a result, this kind of “bottom up” approach—referring to the synthesis of silsesquioxanes with desired physical and chemical properties—is known, justifiable and used. 

### 3.5. Hydrosilylation of Alkenes and Alkynes as Effcient Procedures for the Introduction of Silylalkanyl and Silylalkenyl Moieties tothe Si–O–Si Core

In the chemistry of organosilicon compounds, the hydrosilylation process is of key importance and, in this case, it has a predominant role as a fundamental industrial tool for the synthesis of the aforementioned systems [87,88]. A catalytic insertion of Si–H into multiple bonds, in particular into carbon–carbon unsaturated bonds (C=C and C≡C) including heteroatom, occurs in the presence of transition metal complexes (TM complexes), especially platinum Karstedt’s catalyst = [Pt_2_(dvds)_3_], and nickel, rhodium and iridium based systems in homo- and heterogeneous forms, but also with Lewis acids (e.g., AlCl_3_). The reaction conditions, catalytic systems and reagents’ structures influence the stereo-or even regioselectivity of hydrosilylation leading towards saturated and unsaturated molecular, but also macromolecular organosilicon systems [82]. Hydrosilylation is also a very popular catalytic procedure to anchor organic dyes onto a Si–O–Si core of a cubic T_8_ cage (POSS) or a double-decker (DDSQ) type. 

**Figure 22:**
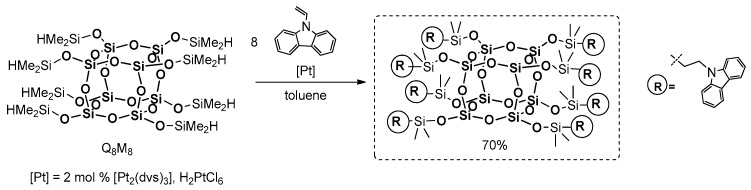
Hydrosilylation reaction towards efficient synthesis of octakis [2-carbazol-9-yl]-ethyldimethylsiloxy]silsesquioxane.

Imae and Kawakami’s group reported a new type of POSS derivative containing carbazole as a photo- and electroactive chromophore [89,90]. The carbazole unit was introduced via hydrosilylation between octa(dimethylsiloxy)silsesquixane (Q_8_M_8_) and 9-vinylcarbazole in the presence of commercially available Karstedt’s ([Pt_2_(dvds)_3_]) as well as Speier’s (H_2_PtCl_6_) catalyst (Figure 22).

The reaction was performed in dry toluene using a small excess of olefin to ensure complete conversion of the reactants, and it led to the formation of the expected product with a 70% yield. Its TGA showed that the product was stable up to ~400 °C in air, and 450 °C in N_2_ (i.e., it exhibited higher stability than its organic analogoue, poly(9-vinylcarbazole) (PVCz), which was also proved by DSC anaysis and was expected by POSS addition). The *T*_g_ = 37 °C was much lower than that of the PVCz (*T*_g_ = 190 °C) due to the flexible spacer between POSS and carbazole. The photophysical parameters were analyzed in solution (THF) and in a solid state, and were compared to their organic couterparts (9-ethylcarbazole (EtCz) and poly(9-vinylcarbazole) (PVCz)). The emmision spectrum of the product (λ_ex_ = 250 nm) showed two peaks, λ_em_ = 353 and 370 nm, which is almost the same as that of the EtCz. Similar compatibility was obtained in terms of quantum yields in air (Φ_p_ = 0.27 and Φ_EtCz_ = 0.30). On the other hand, the PVCz showed weak and broad peaks with a lower quantum yield (Φ_PVCz_ = 0.10) resulting from the formation of excimer. The λ_em_ for solids was more red-shifted towards the wavelength region 350–400 nm and 450 nm, indcating a greater aggregation in crystalline product than that in amorphous material, but the values of the photoluminescence quantum yields were the same. The result suggests that the introduction of the rigid POSS core isolated each of the carbazole units, preventing their aggregation and the formation of excimer, which resulted in a useful concept to apply this new material in a solid-state optelectronic device.

**Figure 23:**
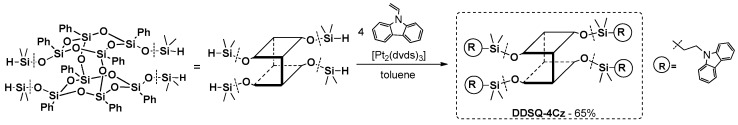
Reaction path towards tetra(carbazole)-substituted DDSQ (DDSQ-4Cz).

Interesting research on the synthesis of carbazole-substituted double-decker-shaped silsesquioxane (DDSQ-4Cz) also obtained via a hydrosilylation reaction (mediated by Karstedt’s catalyst) was presented by Miyashita et al. (Figure 23) [91].

The obtained material’s thermal stability was higher than 355 °C. Photophysical properties of DDSQ-4Cz were disclosed via UV-vis and photoluminescence analysis and compared with its organic analogue (N-vinylcarbazole). The absorption spectrum (in CHCl_3_) revealed of two peaks (λ_ab_ between 325–350 nm) atributted to the π–π* absorption of carbazole, which closely resembled that of N-vinylcarbazole. Interestingly, the photoluminescene spectra of DDSQ-4Cz and N-vinylcarbazole were practically the same (λ_em_ = 350–300 nm), indicating that the four carbazole units were isolated by the rigid core, thereby preventing excimer formation. The authors also demonstrate the obtained material’s potential application in organic electronic diodes. They fabricated an electroluminescent device that showed a maximum brightness of 320 cd m^−2^ at 20 V drive voltage with 40 mA cm^−2^ current density. This proved that DDSQ are promising compounds useful for OLEDs.

**Figure 24:**
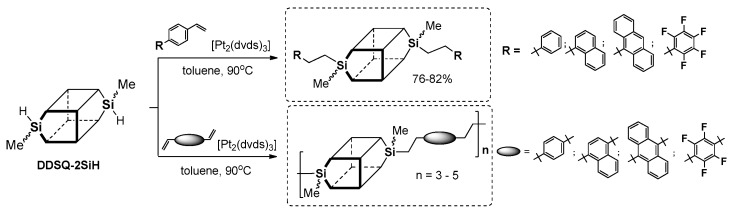
General procedure for the synthesis of molecular and macromolecular aryl-ethyl-double-decker-shaped silsesquioxanes via hydrosilylation.

Dudziec and Marciniec’s groups reported on dihydro-substituted double-decker silsesquioxane (DDSQ-2SiH) efficient modification using Pt-Karstedt’s mediated hydrosilylation reaction protocol with precise reaction time control (FTIR in situ). This facilitated selective formation of a series of molecular as well as macromolecular DDSQ-based systems with ethyl-bridged π-conjugated arenes (Figure 24) [92]. Obtained compounds were verified in terms of their thermal stability (higher than organic couterparts) as well as their photophysics.

A UV-vis analysis of the resultng molecular and macromolecular compounds in comparison to the respective arenes was performed. For the DDSQ-based products, the absorption spectra combined the shape of the DDSQ unit as well as the organic counterpart. For the 1-(4-vinylphenyl)naphthalene derivative, the absorption spectrum showed a 21 nm blue-shifted torn peak at λ_ab_ = 257 and 330 nm, which was different than the basic arene (λ_ab_ = 278 nm). This result may suggest the involvement of Si–O–Si core in the excited state, as has already been reported by Laine and Sellinger for mono- and octa-functionalized silsesquioxanes [47,58]. The photophysical and thermal features could enable the application of these compounds in OLEDs devices. Our group studied the hydrosilylation of olefins and alkynes with silsesquioxanes with one, four and eight reactive Si–H moieties, which constitute a valuable group of precursors used in the formation of photoactive materials [93,94,95,96,97].

**Figure 25:**
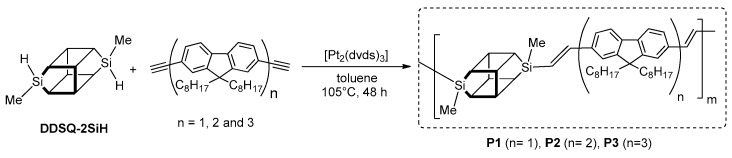
Synthesis of oligomers with the DDSQ unit and oligofluorenes in the main chain via hydrosilylation.

In 2014 Wang, He and Chin demonstrated a practical route to the synthesis of a series of three hybrid polymers containing a periodically interconnected DDSQ unit and oligofluorenes in the main chain [98]. Hydrosilylation was employed to prepare these materials by sequential addition of dihydro-substituted DDSQ (DDSQ-2SiH) and fluorenes: monomer (*n* = 1), dimer (*n* = 2) and trimer (*n* = 3) in the presence of the Karstedt’s catalyst (Figure 25).

The obtained copolymers (P1–P3) had molecular weights of *M_n_* = 9700, (Polydispersity Index) PDI = 2.55; *M_n_* = 18,700, PDI = 1.45; and *M_n_* = 39,300, PDI = 1.34, respectively, and were soluble in common oragnic solvent. TG analyses indicated a high level of thermal resistance in the obtained systems, up to 446 °C. This means that the block copolymer containing DDSQ units displayed an improvement in their thermal stability when compared to neat poly(fluorene) (PF, 417 °C). The authors also demonstrated that the thermal stability of these products was affected by the ratio of DDSQ in the polymer chain, since the DDSQ can lower thermal conductivity and work as a thermal barrier to isolate heat. All polymers were thermoplastic, with significantly higher *T*_g_ (up to 125 °C) than the corresponding PF (*T*_g_ = 55 °C). The optical properties of the hybrid nanocomposites were determined and compared with the chromophores used. The absorption and emission spectra (in solution and solid) were different. According to the literature, conjugated polymers exhibited significant changes in their solid-state spectra because of interchain interaction (e.g., for PF, λ_em_ changed from 459 to 539 nm in the solid state). Surprisingly, in obtained products, no obvious λ_em_ peak shift was observed for solid- and solution-state spectra, while the λ_ab_ of polymer films (solid) was slightly blue-shifted in comparison to those in a solution. These slight shifts were probably due to the aggregation-induced disorder. The authors of the study observed small 4–13 nm red shifts in the PL λ_em_ for solution compared to the solid state, mainly because of the restriction in conformational motion. It is worth mentioning that λ_ab_ and λ_em_ gradually red-shift along with the increase of chain length (i.e., from P1 to P3). It was demonstrated that all the hybrid polymers exhibited a quantum yield higher than 95%, while for the PF it was only 79%. The reason for such high values of P1–P3 quantum yields could be the confinement of excitons within each oligofluorene unit due to the interconnecting DDSQ cage. Thus, the oligofluorene fragments behaved as isolated, not conjugated chromophores. According to the authors, these results suggested that the optical properties of oligofluorenes were faithfully maintained in the proposed final materials. To demonstrate the potential of synthesized materials in organic electronic applications, the authors successfully prepared two OLED devices that maintained color stability and purity when compared to PF.

**Figure 26:**
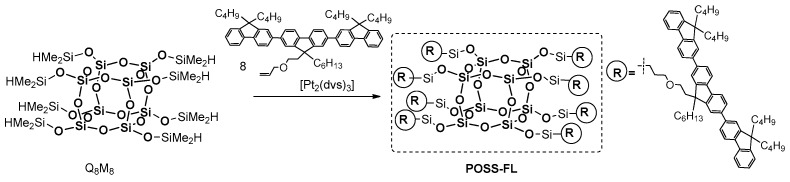
Synthesis of octakis(terfluorene)silsesquioxane via hydrosilylation.

In 2006, Shim et al. demonstrated that a hydrosilylation reaction may be used for the efficient synthesis of POSS-based blue-light electroluminescent (EL) nanoparticles containing terfluorene moieties on each of their eight arms [99]. This material was obtained via the Pt-catalyzed hydrosilylation between commercially available octa(dimethylsiloxy)silsesquioxane T_8_ (Q_8_M_8_) and an allyl-functionalized terfluorene (Figure 26).

The material displayed high thermal stability (*T*_d_^5%^ = 379 °C) and good film-forming properties (tests on a quartz or an indium tin oxide plate). The photophysical analyses were performed in solution (THF) and in solid state. The maximum λ_ab_ in solution was 352 nm (353 nm in a solid state) with PL emission maxima λ_ab_ at 394 and 415 nm (401 and 420 nm in a solid state). These results were compared with those for the organic counterpart poly(dihexylfluorene) and were coincided both in solution and in solid state. This good spectral overlap suggests that POSS–FL can be used as a dopant of blue-light-emissive conjugated polymers, such as polyfluorenes, to increase their quantum efficiencies through energy transference and the isolation of chromophores, as well as in applications requiring electroluminescent nanoparticles. It is worth mentioning that the external quantum efficiency in EL devices of POSS-derivative doped poly(dihexylfluorene) blends were found to be four to eight times higher than that of their organic analogue.

**Figure 27:**
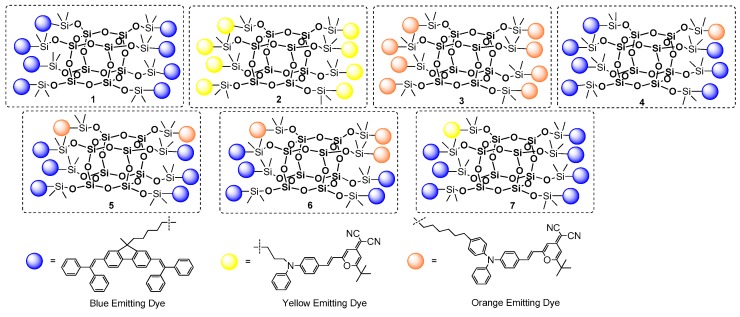
Synthetic hydrosilylation route to T_8_-based systems with mixed organic fluorescent emitters.

Other examples based on the same procedure for anchoring the chromophore part onto the T_8_ core were presented by Lee, Mochizuki and Jabbour group [100]. They used a hydrosilylation process for the synthesis of a series of macromolecular materials composed of an inorganic Q_8_M_8_ core and organic fluorescent emitters covalently attached either monochromatically or in a combination of mixed emitters (POSS with different chromophore systems) (Figure 27).

Hydrosilylation was performed using Karstedt’s catalyst with fixed and controlled reagents’ stoichiometry; however. the final product yields may be not satisfying and may suggest further optimization of the procedure. Monochromatic POSS-emitter products (1–3) were obtained with a 60%, 47% and 61% yield, respectively, while isolated yields of POSS containing a combination of two different emitters (4–7) were not higher than 26%. All compounds were proved to have higher thermal stability than their organic emitters. The most interesting part of the research was disclosed in defining the photophysical properties of obtained compounds. Solution and thin-film photoluminescence spectra were measured and compared for both the free emitters and the modified POSS. It was demonstrated that monochromatic products (1–3) have similar absorbance and emission spectra to their free emitter counterparts both in solution and in a solid state. Thus, using POSS core as a scaffold does not influence the color of the emitters applied. Surprising results were obtained for products bearing more than one type of emitter (4–7). It was found that lower-energy emissions (orange or yellow) dominated in the photoluminescence spectrum for materials 4–7 due to a strong intramolecular energy transference from the neighboring higher-energy blue emitter on the POSS. This high degree of energy transference derives from two major factors: the overlapping of the blue emitter’s emission band with its lower-energy absorption band, and the blue and orange emitters’ short intramolecular distance. Overall, the authors indicated that these materials were easily processable and could be spin-coated from solution for the fabrication of OLED devices. They were also prepared and tested with dopants of selected POSS-based systems. 

**Figure 28:**
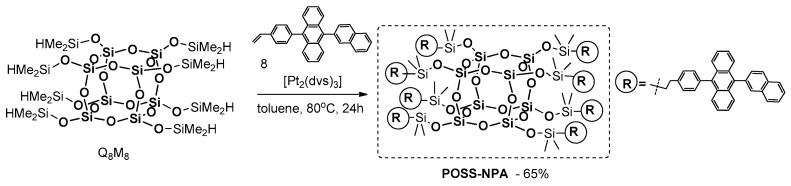
Synthesis of octa(anthracenenaphthyl)silsesquioxane T_8_ via hydrosilylation.

In 2009, Hwang et al. described the synthesis of a similar type of material, i.e., POSS-based electroluminescent nanoparticles, containing anthracenenaphthyl fragments at each of their eight arms [101]. The procedure involves the hydrosilylation of Q_8_M_8_ with vinyl-functionalized 9-naphtalene-2-yl-10-phenyl anthracene in the presence of 2 mol % of Karstedt’s catalyst (Figure 28).

POSS–NPA was analyzed to investigate its thermal and photophysical properties. It was found to be thermally stable up 450 °C, with high *T*_g_ = 154 °C. The UV-vis absorption and PL emission maxima of the final product in solution (chlorobenzene) were found to be 378 and 433 nm, while those characteristics for the product in a solid state were 379 and 464 nm, respectively. These results indicate the aggregation of POSS–NPA molecules and their intermolecular interactions. The authors fabricated an electroluminescent (EL) device that shows blue light emissions.

**Figure 29:**
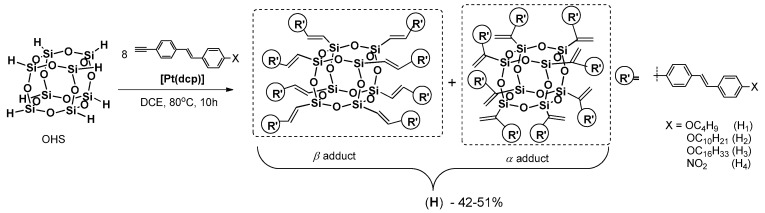
Synthetic pathway for the hydrosilylation of terminal alkynes with OHS.

Xu and Su reported on the synthesis of a new star-type POSS-based molecular hybrid containing stilbene chromophore [102]. These materials were prepared by the hydrosilylation of a series of alkynylstilbenes with octahydridosilsesquioxane (OHS) (Figure 29).

The reaction was performed in refluxing 1,2-dichloroethane (DCE) in the presence of platinum dicyclopentadiene complex [Pt(dcp)], and led to the formation of expected products with isolated yields ranging from 42 to 51%. However, this catalyst seems to have lower selectivity, as in all cases the formation of α and β isomer mixture was detected (α:β = 39–43:57–61). All products exhibited high thermal stability (*T*_d_^5%^ up to 320 °C, *T*_g_ up to 250 °C). The THF solutions of the products were easily cast into films, whereas films of the chromophore could not be obtained in the same conditions. The photophysics were verified and the UV-is spectra (in THF) revealed the presence of strong absorption peaks at λ_ab_ = 336 (H1), 339 (H2), 335 (H3) and 360 (H4) nm. It was also found that alkoxy chain length exerts little influence on the absorption of products H1–3. In the case of H4 the absorption peak was red-shifted, which may be attributable to a stronger intramolecular interaction and formation of the excited state. It was demonstrated that the products (H1–4) and their free emitter counterparts showed nearly the same λ_ab_ and broader absorption bands for the hybrids (H), that may have originated from the σ–π conjugation effect of Si–C=C– in obtained materials. The authors proved that the combination of an inorganic POSS core with stilbene chromophores endowed the resulting materials with novel optical limiting properties and high thermal stability. However, there wano information on how the presence of α and β isomers in H affected these properties.

**Figure 30:**
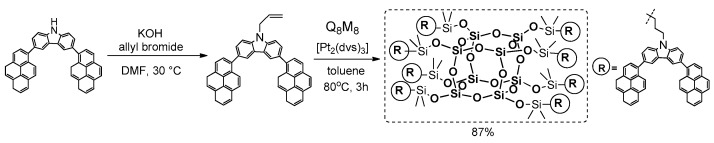
Functionalization of octahydrospherosilicate with *N*-allyl-carbazole derivative.

In 2016, Le et al. developed a new spherosilicate-based compound containing carbazole and pyrene units [103]. This new organic–inorganic amorphous hybrid material was obtained in a two-step reaction involving: (1) synthesis of chromophore using alkylation of *N*-dipyrenylcarbazole with allyl bromide, and (2) hydrosilylation of obtained olefin with Q_8_M_8_ in the presence of Karstedt’s catalyst (Figure 30).

The authors demonstrated that this newly-developed POSS derivative exhibits excellent thermal stability, efficient control of POSS dispersion behavior and good film-forming properties. The photophysical properties of the product were determined and compared with chromophore used in solution (CHCl_3_) and in solid state. The UV-vis spectra of both compounds showed a strong absorption band at λ_ab_ = 281 nm, which can be attributed to the π–π* local electron transition of the carbazole fragment, and a longer-wavelength absorption band at λ_ab_ ca. 349 nm, which corresponds to the π–π* electronic transition of the chromophore backbones. The shape and position of the PL emission spectra was different for the product and chromophore, which can be attributed to the fact that bulky POSS cages reduce the extent of substrate aggregation. This conclusion also supports measurement of PL quantum efficiency (PLQE) of the product (90%), which was much higher than that of the separate chromophore (66%). The results of emission in solid state were interesting, and showed ca. 20 nm red-shift in λ_em_ compared to the solution-phase spectra. This suggests the presence of weak van der Waals forces and intermolecular π–π interactions of adjacent chromophore fragments. Although significantly red-shifted behavior was observed in the film state, the final product exhibited an emission band centered at 454 nm, which was substantially blue-shifted compared to that of the substrate film (463 nm). These observations indicate that the POSS cage inhibits the aggregation of chromophore units to improve their color purity and stability both in solution and the solid state. This also promotes its possible applications in solution-processed optoelectronic devices.

**Figure 31:**
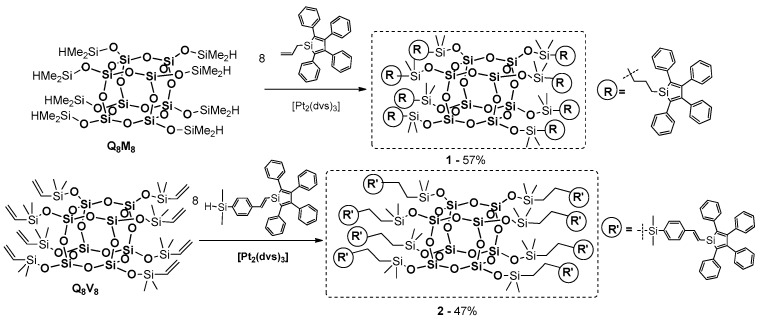
Hydrosilylation-based functionalization of Q_8_M_8_ and Q_8_V_8_ with allyl-tetraphenylsilole and hydrosilylphenyl-tetraphenylsilole.

Li and Xu et al. reported on the synthesis of two organic–inorganic nanohybrid materials obtained by decorating spherosilicate POSS cores with 2,3,4,5-tetraphenylsilole units through a one-step hydrosilylation process (Figure 31) [104]. As presented in Figure 31, Q_8_M_8_ and Q_8_V_8_ were used as synthetic scaffolds and two different silole derivatives were chosen as their reagents. Expected products (**1**, **2**) were isolated with 57% and 47% yields, respectively. Their thermal and photophysical properties were determined along with their utility as potential sensors for the selective detection of nitroaromatic explosives in aqueous media. As expected, thermal stability of these systems was improved (up to 390 °C). Photophysical properties of both products were solvent-dependent. It was observed that product **1** emits weakly at 486 nm in THF and at 496 nm in a mixture of THF/water (1:9), while in 90% water content the photoluminescence intensity is 68-fold higher than in pure THF.

These results suggest that the PL of **1** clearly demonstrates the aggregation-enhanced emission (AEE) of this molecule. Similar emissive behavior was also observed for **2**. For a quantitative comparison, measurements of fluorescence quantum were performed. The absolute fluorescence quantum values of the thin solid films of both products reached as high as 51% and 45%, respectively, meaning they were higher than those of their corresponding parent silole molecules in the solid state. Obtained results proved that the POSS core improved the fluorescence quantum yield of the AEE materials in both the solution and in the aggregated state.

**Figure 32:**
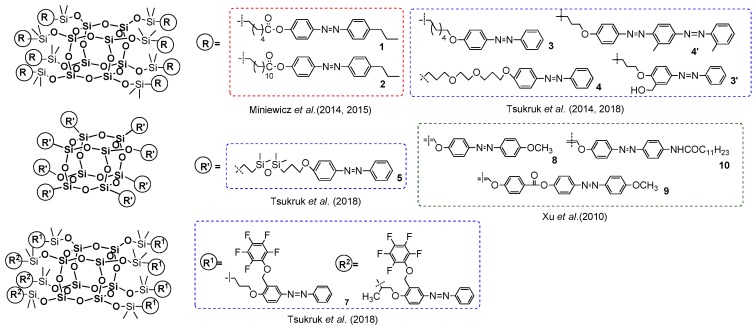
The POSS derivatives with azobenzene-based chromophores obtained by hydrosilylation.

Further examples of the use of silsesquioxane derivatives in hydrosilylation with azo-chromophores in order to form functionalizable POSS nanoparticles have been reported by the Xu, Tsukruk and Miniewicz groups independently [105,106,107,108,109]. The procedures, allowing the synthesis of POSS derivatives with azobenzene mesogenes, have been proposed via the hydrosilylation of respective octahydro-substituted T_8_ silsesquioxanes. Selected structures of final materials (**1**–**10**) are presented in Figure 32.

Xu presented a group of azobenzene-containing POSS-based star-like hybrid functional materials obtained via hydrosilylation octahydridosilsesquioxane with three different azobenzene chromophores containing terminal alkynyl moiety as the reactive fragments [109]. The reactions were carried out in the presence of platinum dicyclopentadiene complex [Pt(dcp)] at 80 °C, using an eight-fold molar excess of olefin relative to silsesquioxane. However, the process was not selective, and expected products were obtained as a mixture of α- and β-adducts with yields ranging from 48% to 54%. Analyzed thermal stability was proved to be higher than of the respective chromophores and suggests its enhancement with anchoring chropomhores onto POSS core. Interestingly, the authors did not observe any sign of melting or glass transition of the products up to the decomposition temperatures, though all substrates were crystalline solids. These amorphous characters of compounds may result from a star-like structure with rigid azobenzene chromophore units protruding from a spherical POSS core in 3D thus minimizing any π–π interactions. The photophysical properties of all products were disclosed using UV-vis and PL analyses, and were compared with their organic analogues. Product 10 and its corresponding azobenzene chromophore displayed nearly the same maximum absorption wavelengths and spectral pattern, indicating that Si–O–Si core has no significant effect on the electronic structure of the final material. The UV-vis spectra of products (8–10) in THF showed strong absorption bands at λ_ab_ = 356, 347 and 365 nm, respectively, which can be assigned to the π–π* local electron transition of the azobenzene fragments. It was also proven that azobenzene moieties influence the λ_ab_ (e.g., the maximum absorption peak of compound **9** shows a 9 nm blue shift compared to that of **8**, which may have resulted from the rigid spacer –PhCOO– unit twisting the planar azobenzene fragment. Analogous results were achieved for spectra recorded in a solid state (λ_ab_ = 360, 347 and 366 nm). This suggests the star-type structure in products enables avoiding the aggregation for azobenzene units in solid state. The authors showed that obtained materials had good film-forming properties, high thermal stability and novel optical limiting properties that could be potentially applied as opto- and electroluminescent materials.

Miniewicz et al. reported on two types of novel nanoparticles based on a POSS surrounded by eight covalently attached units of azobenzene mesogens differing in the length of their aliphatic chains (**1**, **2**) (Figure 32) [105,106]. The compounds were selectively obtained in β isomers using Karsted’s catalyst and obtained with 68.5% and 71% yields, respectively. The UV-vis spectra (in CHCl_3_) of the stable forms of **1** and **2** exhibited features that were characteristic of parent azobenzene molecules in the wavelength range 250–550 nm, as well as a strong π–π * band at λ_ab_ ca. 338 nm (for **1**) and 335 nm (for **2**) and much weaker broad bands with maxima at λ_ab_ ca. 445 nm (for **1**) and 440 nm (for **2**), respectively. These results suggest that, in equilibrium and at room temperature, the azobenzene units are predominantly in their trans configuration. Different absorption spectra were recorded after homogeneously being irradiated by intense broad UV light (360 nm) for 2 min. The disappearance of a band at 335 nm and the increase of intensity of the 440 nm band was observed. This transformation is characteristic of the reversible trans–cis isomerization process in azobenzene units. In further studies, the authors proved that the addition of the POSS compound with mesogenic branches containing azo dye undergo photoisomerization enhanced by UV light.

The Tsukruk group presented a similar type of nanoparticles, but have reported two different approaches to fabricating azo-functionalized POSS compounds via Karstedt’s mediated hydrosilylation. These were carried out in 48 and 72 h, with high 83–94% yields (Figure 32) [107,108]. Firstly, they obtained branched azo–POSS structures based on azo dyes possessing flexible spacers of different chemical natures and lengths between the POSS core and the azobenzene units (**3**–**5**). Secondly, they synthesized the final material by isolating the azobenzene moieties with a constant short spacer between the azo dyes and POSS core (**6**, **7**). The product **7** was obtained as mixture of α and β isomer, and the distribution of these isomers at molecular level was statistical. The higher thermal stability of all systems was proved (for products **3** and **5** the melting point temperatures were detected). The photophysical properties of all products were determined and compared with azo dyes (in solution and solid state). The UV-vis spectra of prepared azobenzene-modified POSS compounds showed two characteristic bands: λ_ab_ at 334–347 nm, which was related to the π–π* transition of the trans-azobenzene form, and λ_ab_ at 427–443 nm, originating from a typical n–π* transition. It was also observed that λ_ab_ peak positions (π–π*) of the initial substrates were identical to their respective POSS products. However, the absorption bands corresponding to π–π* transition in the trans isomers of all products were broader. This may have resulted from partial chromophore–chromophore aggregation of the azobenzene moieties. The UV-vis absorption spectra of the prepared films closely resembled solution spectra. Moreover, the products with the isolated azobenzene fragments (**3**–**5**) revealed a significant decrease in the trans–cis photoisomerization rate in solution as compared to other systems (**6**, **7**). In the solid state, the photoisomerization rates of all products were almost the same. These results indicate that the presence of isolation groups effectively prevents aggregation of substituents attached to the POSS core in films.

A different part of POSS-based frameworks exhibiting interesting photophysical properties are TM complexes, mainly Ir and Pt, that are anchored by functional groups playing the role of a ligand to the silsesquioxane core. The phosphorescent TM complexes used as emitters in OLEDs have attracted much attention because they can utilize singlet as well as triplet excitons through spin-orbital coupling with metal ions [110]. Platinum, and particularly iridium, are the most applicable metals, as they possess high quantum efficiency, brightness, a variety of colors and short excited-state lifetimes, and may be used as an alternative to classical OLEDs or in electrochemical devices, etc. On the other hand, silsesquioxane-based systems using conjugated arenes as substituents exhibit interesting photophysical and also thermal properties. Their incorporation into organic-light-emitting materials prevents aggregation of the organic moieties that lead to emission quenching and/or the lowering of quantum efficiency. The POSS core is suggested to act as a hybrid hole-transport material when compared to traditional hole-transport materials. Additionally, rigid Si–O–Si core improves the thermal stability of the resulting material. These silsesquioxane features have encouraged researchers to consider its application as a scaffold for organic substituents that play role of a ligand to anchor the metal ion and constitute a POSS-based complex. In this part, the proper choice of a coordination sphere for a desired complex (i.e., the presence of respective functional groups at the Si–O–Si core acting as donating groups in ligands, as well as modulating metal ancillary ligands), has evolved over the recent years, and interesting reports on TM complexes anchored onto silsesquioxane cores appeared. Their application in the fabrication of light-emitting devices has been observed previously [110]. The literature review suggests a hydrosilylation reaction (mediated by Karstedt’s complex) as an easy methodology, since the bridge or spacer between the POSS core and the potential ligand play a minor role [111]. This is why the aliphatic connection resulting from hydrosilylation of alkene moiety by Si–H groupis the predominant synthetic procedure that enables formation of POSS-based compounds and their use as potential ligands for TM-complex synthesis.

**Figure 33:**
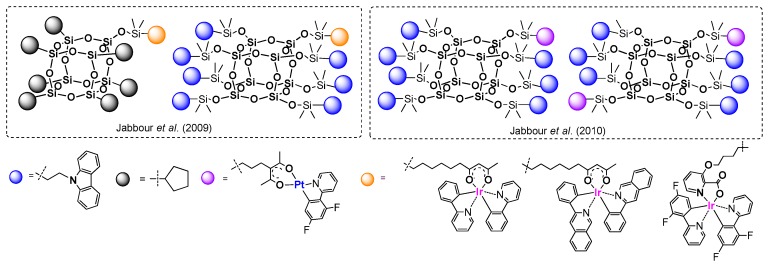
The POSS-based complexes of Ir and Pt obtained by hydrosilylation.

Jabbour et al. pioneered this issue and in 2009 reported on the efficient formation of a series of not only iridium-based, but also platinum-based complexes (Figure 33) [112,113]. The synthetic procedure involved a few stages, which is a general approach for this kind of research. First, the proper design of an organic ligand structure is required. Usually the *N*- and *O*-based ligands are the most favorable, (e.g., based on acetylacetone and pyridine derivatives (2-phenylpyridine, 3-phenylisoquinoline), etc.).

Formation of a respective alkenyl-functionalized linker, placed on the ligand moiety, was used for combining this molecule with the POSS core. Next, the selection and introduction of a proper ancillary ligand to the TM coordination sphere is important. The final part of the research concerns performance of a hydrosilylation reaction to anchor the complex with its functionality onto the POSS core. In these examples, the mono- and octa-substituted POSS was used as a reagent, with the –OMe_2_Si-H moiety as reactive group. In the case of octa-functional systems, one Ir-based complex fragment and one and two Pt-based complex fragments were attached to the silsesquioxane. The rest of the Si–H moieties were reacted with allyl-carbazole. The authors obtained these systems with diverse yields, from very high (92%), to moderate (54–63%) and rather low (~20%), and their photophysical properties were analyzed (CHCl_3_, solid film). In general, the absorption properties of these molecules derive from the absorption of metal-to-ligand charge transfers (MLCT) at λ_ab_ ca. 340–350 nm. The carbazole moieties, together with ligand-centered absorption units, result in shorter wavelengths. New emission peaks at λ_em_ = 465 and 610 nm (green and green-yellow) from the complex with POSS system were red-shifted, especially in solid state for a molecule with two complex moieties in a molecule due to the excimer/aggregate states. Nevertheless, these systems were used in the fabrication of electroluminescent devices that revealed quantum efficiency to be enhanced.

**Figure 34:**
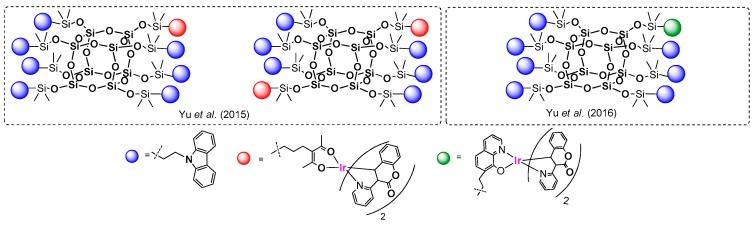
The Ir complexes of spherosilicate obtained through hydrosilylation.

Yu et al. reported on the efficient formation of silsesquioxane-based phosphorescent materials consisting of an emissive Ir(III) complex and carbazole moieties (Figure 34) [114,115].

These systems were based on 3-(pyridin-2-yl)coumarinato N,C^4^) as ancillary ligand and varied the additional ligand that was used to bind the POSS core with allyl group (i.e., 8-hydroxyquinolinolate and acetylacetone derivatives). The authors obtained compounds with one and two Ir moieties anchored to one POSS molecule with 12.5%, 23% and 55% yields, respectively. Their enhanced thermal stability was proven. The photoluminescence spectra of the POSS systems (in DCM and solid state) were obtained. Again, the absorption spectra were derived from carbazole units (λ_ab_ = 360 nm) as well as spin-allowed and spin-forbidden metal-to-ligand charge transfers (MLCT) (λ_ab_ = 400 nm). The emissions of these systems were similar and revealed the presence a weak green emission at λ_em_ = 535 nm, along with a dominant red emission at λ_em_ = 635 nm for 8-hydroxyquinolinolate, a strong green emission at λ_em_ = 530 nm and a dominant green emission λ_em_ = 567 nm. The quenching of the emission within the concentration rise was diminished due to the separation of the chromophores onto the POSS core. These compounds were also successfully applied to the fabrication of electroluminescence devices exhibiting high brightness and higher external quantum efficiencies.

**Figure 35:**
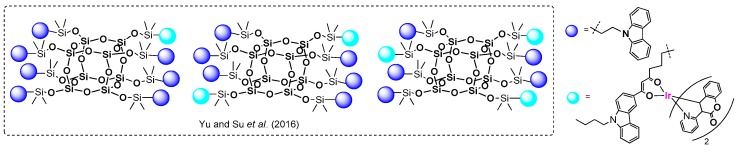
The spherosilicate-based complexes with one, two and three Ir complexes obtained by hydrosilylation.

In 2016, You and Su reported on a synthesis of POSS-based systems containing one, two and three moieties of Ir(III) complex that were also based on 3-(pyridin-2-yl)coumarinato N,C^4^) as an ancillary ligand, and allyl acetylacetone modified with n-Bu-carbazole molecule (Figure 35) [116].

These systems were obtained using the abovementioned general synthetic approach via hydrosilylation, with 26.4%, 14.7% and 6.4% yields, respectively, that may suggest the optimization of reaction conditions for better yields. Obtained compounds were verified in terms of their thermal resistance, which was proven. The absorption and emission spectra were, in general, analogous to the previously reported ones (i.e., absorption derived from the carbazole unit and spin-allowed and -forbidden metal-to-ligand charge-transfer (MLCT) transitions). The emission (λ_ex_ = 430 nm) spectra resembled each other and exhibited mainly green emission at λ_em_ = 530 nm and minor green-yellow at λ_em_ = 570 nm. The results also present a reduction of quenching from Ir-complex moiety interactions due to a bulky POSS core. The electroluminescent device fabricated using the abovementioned compounds revealed an enhanced quantum efficiency that proved its application utility. 

### 3.6. Other Catalytic Reactions leading to the Formation of POSS-Based Systems of Interesting Photophysical Properties

A group of catalytic processes known as “click reactions” has gained a profound interest in (bio-) organic chemistry and also materials chemistry, a term that was successfully introduced into literature by Sharpless in 2001 [117]. In general, these are perceived as mainly one-pot, modular processes, with a wide tolerance to other functional groups that generate minimal and inoffensive byproducts. They are supposed to be stereospecific and are characterized by a high thermodynamic driving force to favor a single product formation with a high yield. Additionally, the process would preferably have mild and simple reaction conditions, along with commercially available reagents to obtain products easily isolated, mainly via crystallization or distillation. Many of these criteria are subjective and depend on a certain type of a process, but several reactions fit the concept better than others, and have been applied in the chemistry of silsesquioxanes [118]:

- [3+2] cycloadditions, such as Huisgen 1,3-dipolar cycloaddition and particularly Cu(I)-catalyzed azide-alkyne cycloaddition (CuAAC)
- thiol-ene coupling reaction (TEC)
- strain-promoted azide-alkyne cycloaddition (SPAAC)
- Diels–Alder reaction
- thiol-Michael addition
- oxime ligation

One of these processes, applied in the synthesis of silsesquioxanes, is the 1,3-dipolar Huisgen cycloaddition of azides to alkynes, to give form 1,2,3-triazoles (CuAAC) [119]. This is a catalytic reaction, mediated mostly by copper(I) salts (but also ruthenium or silver) and reducing agents (e.g., sodium ascorbate) [118,120].

**Figure 36:**
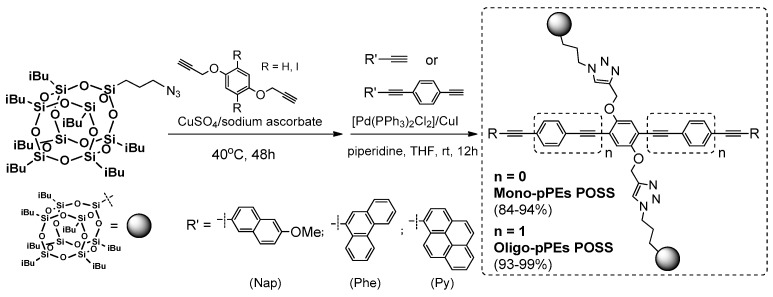
Synthesis of oligophenyl-ethynylenes with heptaisobutylsilsesquioxane as pendant groups via a 1,3-dipolar Huisgen reaction followed by palladium Sonogashira coupling.

A combination of the 1,3-dipolar Huisgen process followed by palladium Sonogashira coupling was used in the synthesis of oligophenyl-ethynylenes with heptaisobutylsilsesquioxane used as pendant groups by Ervithayasuporn et al. (Figure 36) [121].

These two consecutive processes turned out to be efficient methods (84–99% yields) for the synthesis of interesting, highly π-conjugated organic systems, with silsesquioxane units at two independent molecule fragment, of high thermal stability (*T*_d_^5%^ up to 369 °C and exhibited *T*_g_ in a range of 80–197 °C for R’ = Phe oligo-pPE POSS). Their photophysical properties were analyzed in solution (THF) and also in a solid state. While the maxima absorption in solution equaled λ_ab_ = 373–403 nm, the emission spectra (excitation at 350 nm) shift from deep blue to sky blue, and red-shifted (λ_em_ = 409–443 nm) as the amount of aromatic fragments of end-capping molecules increased, as well as with the extended conjugation of in the main chain. The emission spectra of photoluminescence measured in the solid state were strongly shifted towards longer wavelengths (λ_em_ = 470–533), which resulted in interchain orbital interactions referred to aggregation bands. Nevertheless, the extended π-conjugation of oligo-pPEs POSS maintained relatively high photoluminescence quantum efficiencies when compared to mono-pPEs POSS. The authors reported on the preliminary and successful use of mono- and oligo-pPEs POSS (R’ = Py) as active dopants in the fabrication of dye-doped OLED devices.

**Figure 37:**
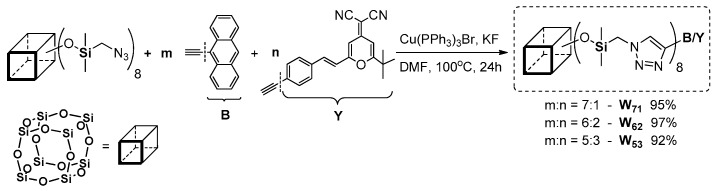
Synthetic route to octa(azidomethylene)spherosilicate derivatives obtained by a 1,3-dipolar Huisgen reaction.

An interesting example of octazido-functional POSS was reported by Xu et al. with two types of functionalities of different stoichiometry obtained via a 1,3-dipolar Huisgen reaction (Figure 37) [122]. The authors used octa(azidomethylene)spherosilicate obtained as an efficient azido-bering reagent [123]. The synthetic procedure was based on a different kind of copper complex, along with the reducing agent (i.e., [Cu(PPh_3_)_3_Br] and KF) that resulted in very high yields of products bearing two types of functionalities B and Y: W_71_, W_62_ and W_53,_ which abbreviations derive from the stoichiometry of the acetylenes applied.

The obtained compounds were thermally more stable (*T*_g_ in a range of 192–273 °C) than their organic counterparts (B and Y). Their photophysical properties were analyzed in comparison to the acetylenes (B and Y) as reference molecules in solution and in a solid state. The absorption and emission spectra (shape, intensity and also λ_em_) of the acetylenes were dependent on the solvent use, and were collected in THF and also THF/water (from a 0 to 90% water fraction). Additionally, the emission spectra of Y were strongly fixed on the amount of water content and were red-shifted ca. 60 nm. As a result, the emission spectra of W_62_ and W_53_ possessing two (W_62_) and three (W_53_) Y groups also exhibited the THF/water mixture emission dependence. The emission spectrum of W_62_ in THF presented two peaks at λ_em_ = 410 and 440 nm, but with the increasing content of water an enhancement of two new red-shifted peaks at ~530 and 570 nm were noted. As a result, the light band was fully covered from 400 to 700 nm, making the realization of a white light emission possible. Interestingly, this feature was preserved in the solid state and enabled its application as dopants in the synthesis of white-light-emitting materials.

**Figure 38:**
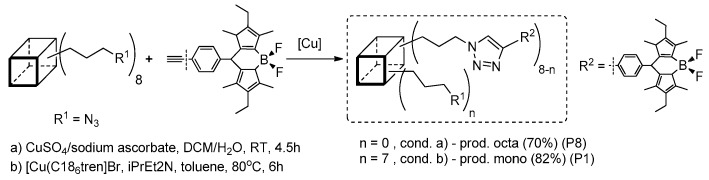
Reaction path towards octa- and mono-BDP derivatives of T_8_ silsesquioxane (BDP–POSS).

López-Arbeloa, García-Moreno and Chiara et al. presented T_8_ silsesquioxane-based dyes that were obtained by a copper(I)-catalyzed 1,3-dipolar cyclo-addition of octakis(3-azidopropyl)octasilsesquioxane with alkyne-substituted 4,4-di-fluoro-4-bora-3a,4a-diaza- *s*-indacene (BDP) chromophore (Figure 38) [124].

The reaction conditions (also reagent stoichiometry) as well as the type of Cu catalyst affected the number of BDP substituents attached to the T_8_ core, and the process was carried out to obtain mono-BDP-substituted and fully (i.e., octa-BDP) substituted product. These two systems, along with their respective BDP analogues (as model compounds), were analyzed in terms of their photophysical properties. P1 and P8 exhibited similar absorption and fluorescence properties (P1: λ_ab_ = 526 nm, λ_em_ = 540 nm; P8: λ_ab_ = 523 nm, λ_em_ = 546 nm) but P8 revealed substantial fluorescence decay that was attributed to the strong π–π interaction of BDP units and formation of H-type or “sandwich” aggregates. Additionally, the fluorescence of P8 was strongly dependent on the type of solvent used (its poor solubility in non-polar media), and acetone was found to be the best. The compounds were analyzed for their lasing properties (in solution and in a solid state) in comparison to the BDP molecular analogues. Interestingly, compound P1 reached a laser efficiency of up to 56% (in the solid matrix) and up to 60% (in ethyl acetate solution) while maintaining a high photostability when compared to the parent BDP model (35%). This proves the high application potential of BDP–POSS-based systems in the formation of new photonic materials as alternative sources for optoelectronic devices. 

**Figure 39:**
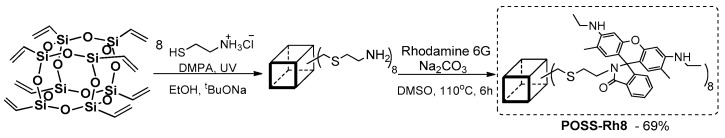
Reaction path towards octa-functionalized silsesquioxane with rhodamine 6G.

An interesting work on the radical thiol–ene reaction (TEC) (mediated via DMPA = 2,2-Dimethoxy-2-phenylacetophenone) of propargyl thiol and octa(vinyl)POSS (OVS) functionalization, and its further ligation with rhodamine 6G, was presented by Mironenko et al. (Figure 39.) [125].

The authors reported on the photophysics of compound and related absorption (λ_ab_ = 532 nm)—emission spectra (λ_em_ = 554 nm) that were pH dependent and increased in intensity with a decrease in pH value. Due to their problems with solubility, an increase of intensity was noted for higher-content ethanol in the water/ethanol solution. This compound was tested as a fluorescent chemosensor for a variety of metals, and the results indicated a positive response for Au^3+^ ion (50:50 water/ethanol, pH = 1 and molar concentration of rhodamine units of 10^−5^ M). Under these conditions, there was a strong fluorescence emission enhancement. As a result, POSS–Rh8 may be used as selective fluorescent chemosensor for the selective determination of Au(3+) ions in aqueous media.

One of the reactions applied in the chemistry of silsesquioxanes was free radical copolymerization leading to hybrid materials that are more amenable to solution techniques (e.g., spin-coating that results in large-area device fabrication). On the other hand, the phosphorescence of metal complexes, especially iridium or platinum, and their use in specific types of OLED devices was reported [110]. However, these complexes may suffer from self-quenching in the solid due to interaction aggregation. This may be improved by anchoring them onto the Si–O–Si core (described in Section 3.4). The idea of grafting of non-conjugated polymers (as a transparent matrix) with the metal complex covalently attached to the polymer backbone was studied. In this scenario, solubility would be additionally improved in comparison to the conjugated counterparts, thus facilitating application. 

**Figure 40:**
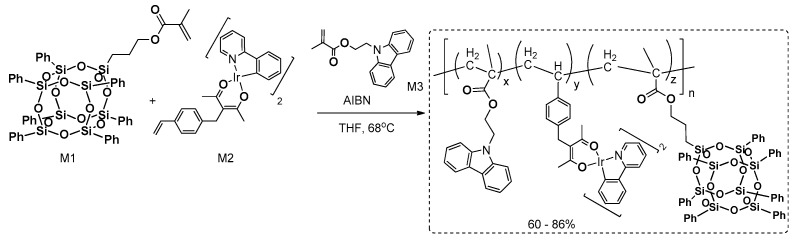
The synthetic route of the copolymer-based Ir complex.

An interesting paper of Ling and Lin et al. reported on the efficient synthesis of poly(methylmethacrylate) via the AIBN (2,2′-Azobis(2-methylpropionitrile))-mediated radical copolymerization of two types of methylmethacrylates (bearing mono(propyl)heptaphenyl(silsesquioxne) = M3, carbazole unit = M1, with styryl-based iridium complex = M2) used in different molar ratio (POSS from 2 to 8 mol %) and resulting in copolymeric systems (Figure 40) [126]. Depending on the specific ratio of comonomers, the molecular weights varied (*M_n_* = 5100–19,400 g/mol, suggesting formation of other oligomeric systems), with PDI from 1.4 to 2.2.

The obtained materials were characterized in terms of their absorption emission properties, comparing the results with analyses for each monomer M2, M3 along with the copolymer without POSS (i.e., M2 + M3 and final ternary copolymers M1 + M2 + M3 with 2–8 mol % POSS contribution). The absorption spectra of copolymers in DCM showed peaks at λ_ab_ = 237, 261, 294, 328 and 342 nm, which came from the carbazole segment and iridium complex moiety. The photoluminescence spectra (λ_ex_ = 365 nm) in solution exhibited blue and green emission bands λ_em_ ca. 420 and 435 nm (derived from the host carbazole unit M1), with additional green emission bands at λ_em_ ca. 530 nm (derived from the guest iridium complex fragment M2). The band at 530 nm was enhanced with the increase of iridium complex content from 0.5 to 5.0 mol % while the bands around 420 and 435 nm decreased in intensity. There was energy transference suggested by the host (donor) carbazole moiety to the guest (acceptor) iridium complex moiety, which is also dependent on the content of Ir units along with the POSS moiety. However, the ternary copolymer with 5 mol % or iridium units and 2 mol % of POSS exhibited a significant enhancement of the green emission that may have indicated the separation of Ir complex units, preventing their aggregation, so that the concentration-quenching was restrained in the solution. In a solid state, the emission spectra presented only one little red-shifted green emission band (up to ~10 nm) but there was an interesting enhancement of the spectra intensity for the copolymer with 0.5 mol % of Ir complex content, meaning that the POSS unit prevented the aggregation of Ir units more effectively—a phenomenon that was more intensive with an increase in POSS content. The quantum efficiencies for these systems in a solid state were also higher, up to 52% (when compared to binary copolymers M2 + M3, Φ = 7–24%), which suggests that incorporation of POSS units enhances luminescence profoundly. Thermal resistance was also improved. These aspects make the obtained materials definite candidates for OLED devices.

## 4. Conclusions

The continuous demand for novel hybrid materials of specific applications inspires researchers to develop new synthetic procedures in modular and efficient ways. In this review, we have concentrated on the last decade, most effective routes for synthesizing functionalized polysilsesquioxanes to be employed in POSS-based photoactive materials and/or their precursors.

- The first and essential step towards the synthesis of the abovementioned frameworks is the stoichiometric, hydrolytic condensation of prefunctionalized chloro- or alkoxysilanes, to constitute a basic silsesquioxane core with respective reactivity. These systems are, in turn, the basis for further modification (i.e., the introduction of highly functionalized organic moieties of complex architecture, usually and profoundly via catalytic methods).
- Heck coupling (HC) reactions catalyzed by palladium complex proceeding between C=C bond (especially SiCH=CH_2_) and aryl halides is a reaction very commonly applied for the synthesis of π-conjugated arene systems and related optoelectronic compounds. The respective Sonogashira coupling (i.e., a reaction of aryl halides with terminal alkynes) is much less frequently used.
- Olefin cross-metathesis (CM), as well as silylative coupling (SC) reactions, enable the formation of silylalkenyl moieties, which may be obtained in the presence of Grubbs ruthenium carbene (Ru = CHPh) (CM) or Ru–H/Si (SC) catalyst. These are the other generally effective and highly selective methods. Also, Ge–HC=CH_2_ group in germasilsesquioxane undergo effective cross-metathetic transformation.
- Hydrosilylation of olefins and/or acetylenes is performed predominantly with hydrospherosilicate and catalyzed by TM (usually Pt) complex, and is prevalent reaction that can be efficient step towards the synthesis of optoelectronic materials. Additionally, when dihydro-substituted silsesquioxane (especially DDSQ) is applied as a reagent, the formation of respective macromolecular (polymeric) material with a Si–O–Si core embedded can be also obtained.
- In the last part of this review, other catalytic processes leading to the functionalized silsesquioxane of potent optoelectronic materials was collected.

The methodology presented for the synthesis of POSS-based photoactive materials involves mono-, octa-functional cubic or double-decker silsesquioxanes. As was illustrated, the crucial aspect is to design a certain organic dye of respective photophysical properties (color of emitted light) and to anchor it on to the POSS core using the proper reaction procedure, which is dependent on the type of functional group at the Si–O–Si core. According to the perspective presented by experts in the chemistry of POSS-based photoactive compounds, this is now the next step required for the development of attractive and selective routes leading to multi-functional silsesquioxane derivatives with a markedly broadened application.

## References

[1] POSS-Hybrid Plastics. Registered Trademark. https://hybridplastics.com/.

[2] Hartmann-Thompson C. (2011). Applications of Polyhedral Oligomeric Silsesquioxanes.

[3] Cordes D.B., Lickiss P.D., Rataboul F. (2010). Recent developments in the chemistry of cubic polyhedral oligosilsesquioxanes. Chem. Rev..

[4] Yoshizawa K., Morimoto Y., Watanabe K., Ootake N. (2008). Silsesquioxane derivative and process for producing the same. U.S. Patent.

[5] Morimoto Y., Watanabe K., Ootake N., Inagaki J., Yoshida K., Ohguma K. (2008). Silsesquioxane derivative and production process for the same. U.S. Patent.

[6] Dudziec B., Marciniec B. (2017). Double-decker Silsesquioxanes: Current Chemistry and Applications. Curr. Org. Chem..

[7] Li G., Wang L., Ni H., Pittman C.U. (2001). Polyhedral Oligomeric Silsesquioxane (POSS) Polymers and Copolymers: A Review. J. Inorg. Organomet. Polym..

[8] Zhou H., Ye Q., Xu J. (2017). Polyhedral oligomeric silsesquioxane-based hybrid materials and their applications. Mater. Chem. Front..

[9] Laine R.M. (2005). Nanobuilding blocks based on the [OSiO1.5]x (x = 6, 8, 10) octasilsesquioxanes. J. Mater. Chem..

[10] Ayandele E., Sarkar B., Alexandridis P. (2012). Polyhedral Oligomeric Silsesquioxane (POSS)-Containing Polymer Nanocomposites. Nanomaterials.

[11] Ye Q., Zhou H., Xu J. (2016). Cubic polyhedral oligomeric silsesquioxane based functional materials: Synthesis, assembly, and applications. Chem. Asian J..

[12] Zhang W., Müller A. (2013). Architecture, self-assembly and properties of well-defined hybrid polymers based on polyhedral oligomeric silsequioxane (POSS). Prog. Polym. Sci..

[13] Tanaka K., Chujo Y. (2012). Advanced functional materials based on polyhedral oligomeric silsesquioxane (POSS). J. Mater. Chem..

[14] Quadrelli E.A., Basset J.M. (2010). On silsesquioxanes’ accuracy as molecular models for silica-grafted complexes in heterogeneous catalysis. Coord. Chem. Rev..

[15] Haxton K.J., Cole-Hamilton D.J., Morris R.E. (2004). The structure of phosphine-functionalised silsesquioxane-based dendrimers: A molecular dynamics study. Dalt. Trans..

[16] Crowley C., Klanrit P., Butler C.R., Varanou A., Hynds R.E., Chambers R.C., Seifalian A.M., Birchall M.A., Janes S.M. (2016). Biomaterials Surface modi fi cation of a POSS-nanocomposite material to enhance cellular integration of a synthetic bioscaffold Manuela Plat e. Biomaterials.

[17] John Ł. (2018). Selected developments and medical applications of organic–inorganic hybrid biomaterials based on functionalized spherosilicates. Mater. Sci. Eng. C.

[18] Chan K.L., Sonar P., Sellinger A. (2009). Cubic silsesquioxanes for use in solution processable organic light emitting diodes (OLED). J. Mater. Chem..

[19] Yang Z., Gao M., Wu W., Yang X., Sun X.W., Zhang J., Wang H.C., Liu R.S., Han C.Y., Yang H. (2018). Recent advances in quantum dot-based light-emitting devices: Challenges and possible solutions. Mater. Today.

[20] Liu B., Nie H., Zhou X., Hu S., Luo D., Gao D., Zou J., Xu M., Wang L., Zhao Z. (2016). Manipulation of charge and exciton distribution based on blue aggregation-induced emission fluorophors: A novel concept to achieve high-performance hybrid white organic light-emitting diodes. Adv. Funct. Mater..

[21] Luo D., Yang Y., Xiao Y., Zhao Y., Yang Y., Liu B. (2017). Regulating Charge and Exciton Distribution in High-Performance Hybrid White Organic Light-Emitting Diodes with n-Type Interlayer Switch. Nano-Micro Lett..

[22] Luo D., Chen Q., Gao Y., Zhang M., Liu B. (2018). Extremely Simplified, High-Performance, and Doping-Free White Organic Light-Emitting Diodes Based on a Single Thermally Activated Delayed Fluorescent Emitter. ACS Energy Lett..

[23] Liu B.-Q., Wang L., Gao D.-Y., Zou J.-H., Ning H.-L., Peng J.-B., Cao Y. (2016). Extremely high-efficiency and ultrasimplified hybrid white organic light-emitting diodes exploiting double multifunctional blue emitting layers. Light Sci. Appl..

[24] Sellinger A., Laine R.M. (2003). Organic-Inorganic Hybrid Light Emitting Devices (HLED). U.S. Patent.

[25] Lucenti E., Botta C., Cariati E., Righetto S., Scarpellini M., Tordin E., Ugo R. (2013). New organic-inorganic hybrid materials based on perylene diimide-polyhedral oligomeric silsesquioxane dyes with reduced quenching of the emission in the solid state. Dye. Pigment..

[26] Clarke D., Mathew S., Matisons J., Simon G., Skelton B.W. (2012). Synthesis and characterization of a range of POSS imides. Dye. Pigment..

[27] Du F., Bao Y., Liu B., Tian J., Li Q., Bai R. (2013). POSS-containing red fluorescent nanoparticles for rapid detection of aqueous fluoride ions. Chem. Commun..

[28] Liu Y., Wang K.-R., Guo D.-S., Jiang B.-P. (2009). Supramolecular Assembly of Perylene Bisimide with *β*-Cyclodextrin Grafts as a Solid-State Fluorescence Sensor for Vapor Detection. Adv. Funct. Mater..

[29] Du F., Tian J., Wang H., Liu B., Jin B., Bai R. (2012). Synthesis and Luminescence of POSS-Containing Perylene Bisimide-Bridged Amphiphilic Polymers. Macromolecules.

[30] Asuncion M.Z., Laine R.M. (2010). Fluoride rearrangement reactions of polyphenyl- and polyvinylsilsesquioxanes as a facile route to mixed functional phenyl, vinyl T_10_ and T_12_ Silsesquioxanes. J. Am. Chem. Soc..

[31] Ronchi M., Sulaiman S., Boston N.R., Laine R.M. (2010). Fluoride catalyzed rearrangements of polysilsesquioxanes, mixed Me, vinyl T_8_, Me, vinyl T_10_ and T_12_ cages. Appl. Organomet. Chem..

[32] Boatz J.A., Rzsp A., Mabry J.M., Rzsm A., Mitchell C. (2008). Structural Investigation of Fluoridated POSS Cages Using Ion Mobility Mass Spectrometry and Molecular Mechanics Preprint. Chem. Mater..

[33] Kunthom R., Piyanuch P., Wanichacheva N., Ervithayasuporn V. (2018). Cage-like silsesequioxanes bearing rhodamines as fluorescence Hg2+ sensors. J. Photochem. Photobiol. A Chem..

[34] Zhou H., Ye Q., Wu X., Song J., Cho C.M., Zong Y., Tang B.Z., Hor T.S.A., Yeow E.K.L., Xu J. (2015). A thermally stable and reversible microporous hydrogen-bonded organic framework: Aggregation induced emission and metal ion-sensing properties. J. Mater. Chem. C.

[35] Çakal D., Ertan S., Cihaner A., Önal A.M. (2019). Electrochemical and optical properties of substituted phthalimide based monomers and electrochemical polymerization of 3,4-ethylenedioxythiophene-polyhedral oligomeric silsesquioxane (POSS) analogue. Dye. Pigment..

[36] Ertan S., Kaynak C., Cihaner A. (2017). A platform to synthesize a soluble poly(3,4-ethylenedioxythiophene) analogue. J. Polym. Sci. Part A Polym. Chem..

[37] Ertan S., Cihaner A. (2018). Improvement of optical properties and redox stability of poly(3,4-ethylenedioxythiophene). Dye. Pigment..

[38] Huang H., Lin H., Kershaw S.V., Susha A.S., Choy W.C.H., Rogach A.L. (2016). Polyhedral Oligomeric Silsesquioxane Enhances the Brightness of Perovskite Nanocrystal-Based Green Light-Emitting Devices. J. Phys. Chem. Lett..

[39] Huang H., Chen B., Wang Z., Hung T.F., Susha A.S., Zhong H., Rogach A.L. (2016). Water resistant CsPbX3nanocrystals coated with polyhedral oligomeric silsesquioxane and their use as solid state luminophores in all-perovskite white light-emitting devices. Chem. Sci..

[40] Beletskaya I.P., Cheprakov A.V. (2000). Heck reaction as a sharpening stone of palladium catalysis. Chem. Rev..

[41] Liras M., Pintado-Sierra M., Amat-Guerri F., Sastre R. (2011). New BODIPY chromophores bound to polyhedral oligomeric silsesquioxanes (POSS) with improved thermo- and photostability. J. Mater. Chem..

[42] Liu Y., Yang W., Liu H. (2015). Azobenzene-functionalized cage silsesquioxanes as inorganic-organic hybrid, photoresponsive, nanoscale, building blocks. Chem. A Eur. J..

[43] Sellinger A., Tamaki R., Laine R.M., Ueno K., Tanabe H., Williams E., Jabbour G.E. (2005). Heck coupling of haloaromatics with octavinylsilsesquioxane: solution processable nanocomposites for application in electroluminescent devices. Chem. Commun. (Camb)..

[44] Chanmungkalakul S., Ervithayasuporn V., Hanprasit S., Masik M., Prigyai N., Kiatkamjornwong S. (2017). Silsesquioxane cages as fluoride sensors. Chem. Commun..

[45] Chanmungkalakul S., Ervithayasuporn V., Boonkitti P., Phuekphong A., Prigyai N., Kladsomboon S., Kiatkamjornwong S. (2018). Anion identification using silsesquioxane cages. Chem. Sci..

[46] Lo M.Y., Zhen C., Lauters M., Ghassan J.E., Sellinger A. (2007). Organic−Inorganic Hybrids Based on Pyrene Functionalized Octavinylsilsesquioxane Cores for Application in OLEDs. J. Am. Chem. Soc..

[47] Yang X.H., Giovenzana T., Feild B., Jabbour G.E., Sellinger A. (2012). Solution processeable organic–inorganic hybrids based on pyrene functionalized mixed cubic silsesquioxanes as emitters in OLEDs. J. Mater. Chem..

[48] Wang S., Guang S., Xu H., Ke F. (2015). Controllable preparation and properties of active functional hybrid materials with different chromophores. RSC Adv..

[49] Ke F., Wang S., Guang S., Liu Q., Xu H. (2015). Synthesis and properties of broad-band absorption POSS-based hybrids. Dye. Pigment..

[50] Pu K.Y., Li K., Zhang X., Liu B. (2010). Conjugated oligoelectrolyte harnessed polyhedral oligomeric silsesquioxane as light-up hybrid nanodot fortwo-photon fluorescence imaging of cellular nucleus. Adv. Mater..

[51] Ding D., Pu K.-Y., Li K., Liu B. (2011). Conjugated oligoelectrolyte-polyhedral oligomeric silsesquioxane loaded pH-responsive nanoparticles for targeted fluorescence imaging of cancer cell nucleus. Chem. Commun..

[52] Sun R., Feng S., Wang D., Liu H. (2018). Fluorescence-Tuned Silicone Elastomers for Multicolored Ultraviolet Light-Emitting Diodes: Realizing the Processability of Polyhedral Oligomeric Silsesquioxane-Based Hybrid Porous Polymers. Chem. Mater..

[53] Sun R., Huo X., Lu H., Feng S., Wang D., Liu H. (2018). Recyclable fluorescent paper sensor for visual detection of nitroaromatic explosives. Sens. Actuators B Chem..

[54] Brick C.M., Tamaki R., Kim S., Asuncion M.Z., Roll M., Nemoto T., Ouchi Y., Chujo Y., Laine R.M. (2005). Spherical, Polyfunctional Molecules Using Poly (bromophenylsilsesquioxane) s as Nanoconstruction Sites. Macromolecules.

[55] Roll M.F., Asuncion M.Z., Kampf J., Laine R.M. (2008). para-Octaiodophenylsilsesquioxane, [p-IC6H4SiO1.5]8, a Nearly Perfect Nano-Building Block. ACS Nano.

[56] Roll M.F., Kampf J.W., Kim Y., Yi E., Laine R.M. (2010). Nano Building Blocks via Iodination of [PhSiO 1.5] n, Forming High-Surface-Area, Thermally Stable, Microporous Materials via Thermal Elimination of I 2. J. Am. Chem. Soc..

[57] Brick C.M., Ouchi Y., Chujo Y., Laine R.M. (2005). Robust Polyaromatic Octasilsesquioxanes from Polybromophenylsilsesquioxanes, Br x OPS, via Suzuki Coupling. Macromolecules.

[58] Laine R.M., Sulaiman S., Brick C., Roll M., Tamaki R., Asuncion M.Z., Neurock M., Filhol J.-S., Lee C.-Y., Zhang J. (2010). Synthesis and Photophysical Properties of Stilbeneoctasilsesquioxanes. Emission Behavior Coupled with Theoretical Modeling Studies Suggest a 3-D Excited State Involving the Silica Core. J. Am. Chem. Soc..

[59] Roll M.F., Mathur P., Takahashi K., Kampf J.W., Laine R.M. (2011). [PhSiO1.5]8 promotes self-bromination to produce [o-BrPhSiO1.5]8: further bromination gives crystalline [2,5-Br2PhSiO1.5]8 with a density of 2.32 g cm−3 and a calculated refractive index of 1.7 or the tetraicosa bromo compound [Br3PhSiO1.5]8. J. Mater. Chem..

[60] Sulaiman S., Zhang J., Goodson T., Laine R.M. (2011). Synthesis, characterization and photophysical properties of polyfunctional phenylsilsesquioxanes: [o-RPhSiO1.5]8, [2,5-R2PhSiO1.5]8, and [R3PhSiO1.5]8. compounds with the highest number of functional units/unit volume. J. Mater. Chem..

[61] Jung J.H., Furgal J.C., Clark S., Schwartz M., Chou K., Laine R.M. (2013). Beads on a Chain (BoC) Polymers with Model Dendronized Beads. Copolymerization of [(4-NH2C6H4SiO1.5)6(IPhSiO1.5)2] and [(4-CH3OC6H4SiO1.5)6(IPhSiO1.5)2] with 1,4-Diethynylbenzene (DEB) Gives Through-Chain, Extended 3-D Conjugation in the Excited State Tha. Macromolecules.

[62] Hwan Jung J., Furgal J.C., Goodson T., Mizumo T., Schwartz M., Chou K., Vonet J.F., Laine R.M. (2012). 3-D molecular mixtures of catalytically functionalized [vinylSiO 1.5] 10/[vinylSiO 1.5] 12. Photophysical characterization of second generation derivatives. Chem. Mater..

[63] Furgal J.C., Jung J.H., Clark S., Goodson T., Laine R.M. (2013). Beads on a Chain (BoC) Phenylsilsesquioxane (SQ) Polymers via F− Catalyzed Rearrangements and ADMET or Reverse Heck Cross- coupling Reactions: Through Chain, Extended Conjugation in 3-D with Potential for Dendronization. Macromolecules.

[64] Chinchilla R., Nájera C. (2007). The Sonogashira reaction: A booming methodology in synthetic organic chemistry. Chem. Rev..

[65] Asuncion M.Z., Roll M.F., Laine R.M. (2008). Octaalkynylsilsesquioxanes, Nano Sea Urchin Molecular Building Blocks for 3-D-Nanostructures. Macromolecules.

[66] Gon M., Sato K., Tanaka K., Chujo Y. (2016). Controllable intramolecular interaction of 3D arranged π-conjugated luminophores based on a POSS scaffold, leading to highly thermally-stable and emissive materials. RSC Adv..

[67] Pietraszuk C., Pawluć P., Marciniec B., Grubbs R., O’Leary D.J. (2015). Handbook of Metathesis. Handbook on Metathesis. Vol. 2: Applications in Organic Synthesis.

[68] Feher F.J., Soulivong D., Eklund A.G., Wyndham K.D. (1997). Cross-metathesis of alkenes with vinyl-substituted silsesquioxanes and spherosilicates: A new method for synthesizing highly-functionalized Si/O frameworks. Chem. Commun..

[69] Cheng G., Vautravers N.R., Morris R.E., Cole-hamilton D.J. (2008). Synthesis of functional cubes from octavinylsilsesquioxane (OVS). Org. Biomol. Chem..

[70] Żak P., Dudziec B., Kubicki M., Marciniec B. (2014). Silylative Coupling versus Metathesis-Efficient Methods for the Synthesis of Difunctionalized Double-Decker Silsesquioxane Derivatives. Chem. A Eur. J..

[71] Sulaiman S., Bhaskar A., Zhang J., Guda R., Goodson T., Laine R.M. (2008). Molecules with perfect cubic symmetry as nanobuilding blocks for 3-D assemblies. Elaboration of octavinylsilsesquioxane. Unusual luminescence shifts may indicate extended conjugation involving the silsesquioxane core. Chem. Mater..

[72] Vautravers N.R., André P., Slawin A.M.Z., Cole-Hamilton D.J. (2009). Synthesis and characterization of photoluminescent vinylbiphenyl decorated polyhedral oligomeric silsesquioxanes. Org. Biomol. Chem..

[73] Vautravers N.R., André P., Cole-Hamilton D.J. (2009). Fluorescence activation of a polyhedral oligomeric silsesquioxane in the presence of reducing agents. J. Mater. Chem..

[74] Żak P., Marciniec B., Majchrzak M., Pietraszuk C. (2011). Highly effective synthesis of vinylfunctionalised cubic silsesquioxanes. J. Organomet. Chem..

[75] Araki H., Naka K. (2012). Syntheses and properties of dumbbell-shaped POSS derivatives linked by luminescent pi-conjugated units. J. Polym. Sci. Part A Polym. Chem..

[76] Żak P., Pietraszuk C., Marciniec B., Spólnik B., Danikiewicz W. (2009). Efficient functionalisation of cubic monovinylsilsesquioxanes via cross-metathesis and silylative coupling with olefins in the presence of ruthenium complexes. Adv. Synth. Catal..

[77] Furgal J.C., Jung J.H., Goodson T., Laine R.M. (2013). Analyzing Structure—Photophysical Property Relationships for Isolated T_8_, T_10_, and T_12_ Stilbenevinylsilsesquioxanes. J. Am. Chem. Soc..

[78] Cabrera-González J., Ferrer-Ugalde A., Bhattacharyya S., Chaari M., Teixidor F., Gierschner J., Núñez R. (2017). Fluorescent carborane-vinylstilbene functionalised octasilsesquioxanes: Synthesis, structural, thermal and photophysical properties. J. Mater. Chem. C.

[79] Marciniec B., Pietraszuk C. (1997). Silylation of Styrene with Vinylsilanes Catalyzed by RuCl (SiR 3)(CO)(PPh 3) 2 and RuHCl (CO)(PPh 3) 3. Organometallics.

[80] Marciniec B., Pietraszuk C. (1995). Insertion of Vinylsilane into the Ruthenium-Silicon Bond-Direct Evidence. J. Chem. Soc. Chem. Commun..

[81] Wakatsuki Y., Yamazaki H., Nakano M., Yamamoto Y. (1991). Ruthenium-catalysed disproportionation between vinylsilanes and mono-substituted alkenes via silyl group transfer. J. Chem. Soc. Chem. Commun..

[82] Marciniec B. (2007). Catalytic Coupling of sp^2^- and sp-Hybridized Carbon–Hydrogen Bonds with Vinylmetalloid Compounds. Acc. Chem. Res..

[83] Frackowiak D., Żak P., Spólnik G., Pyziak M., Marciniec B. (2015). New Vinylgermanium Derivatives of Silsesquioxanes and Their Ruthenium Complexes - Synthesis, Structure, and Reactivity. Organometallics.

[84] Żak P., Delaude L., Dudziec B., Marciniec B. (2018). N-Heterocyclic carbene-based ruthenium-hydride catalysts for the synthesis of unsymmetrically functionalized double-decker silsesquioxanes. Chem. Commun..

[85] Żak P., Majchrzak M., Wilkowski G., Dudziec B., Dutkiewicz M., Marciniec B. (2016). Synthesis and characterization of functionalized molecular and macromolecular double-decker silsesquioxane systems. RSC Adv..

[86] Żak P., Frąckowiak D., Grzelak M., Bołt M., Kubicki M., Marciniec B. (2016). Olefin Metathesis of Vinylgermanium Derivatives as Method for the Synthesis of Functionalized Cubic and Double-Decker Germasilsesquioxanes. Adv. Synth. Catal..

[87] Marciniec B., Maciejewski H., Pietraszuk C., Pawluć P., Marciniec B. (2009). Hydrosilylation: A Comprehensive Review on Recent Advances.

[88] Troegel D., Stohrer J. (2011). Recent advances and actual challenges in late transition metal catalyzed hydrosilylation of olefins from an industrial point of view. Coord. Chem. Rev..

[89] Imae I., Kawakami Y., Ooyama Y., Harima Y. (2007). Solid state photoluminescence property of a novel poss-based material having carbazole. Macromol. Symp..

[90] Imae I., Kawakami Y. (2005). Unique photoluminescence property of a novel perfectly carbazole-substituted POSS. J. Mater. Chem..

[91] Kohri M., Matsui J., Watanabe A., Miyashita T. (2010). Synthesis and Optoelectronic Properties of Completely Carbazole-substituted Double-decker-shaped Silsesquioxane. Chem. Lett..

[92] Walczak M., Januszewski R., Majchrzak M., Kubicki M., Dudziec B., Marciniec B. (2017). The unusual cis- and trans-architecture of dihydrofunctional double-decker shaped silsesquioxane–design and construction of its ethyl bridged π-conjugated arene derivatives. New J. Chem..

[93] Stefanowska K., Franczyk A., Szyling J., Pyziak M., Pawluć P., Walkowiak J. (2018). Selective hydrosilylation of alkynes with octaspherosilicate (HSiMe2O)8Si8O12. Chem. Asian J..

[94] Walczak M., Stefanowska K., Franczyk A., Walkowiak J., Wawrzyńczak A., Marciniec B. (2018). Hydrosilylation of alkenes and alkynes with silsesquioxane (HSiMe2O)(i-Bu)7Si8O12 catalyzed by Pt supported on a styrene-divinylbenzene copolymer. J. Catal..

[95] Dutkiewicz M., Maciejewski H., Marciniec B., Karasiewicz J. (2011). New fluorocarbofunctional spherosilicates: Synthesis and characterization. Organometallics.

[96] Walczak M., Januszewski R., Franczyk A., Marciniec B. (2018). Synthesis of monofunctionalized POSS through hydrosilylation. J. Organomet. Chem..

[97] Duszczak J., Mituła K., Januszewski R., Żak P., Dudziec B., Marciniec B. (2019). Highly efficient route for the synthesis of a novel generation of tetraorganofunctional double-decker type of silsesquioxanes. ChemCatChem.

[98] Chi H., Lim S.L., Wang F., Wang X., He C., Chin W.S. (2014). Pure blue-light emissive poly(oligofluorenes) with bifunctional POSS in the main chain. Macromol. Rapid Commun..

[99] Cho H.J., Hwang D.H., Lee J.I.J., Jung Y.K., Park J.H., Lee J.I.J., Lee S.K., Shim H.K. (2006). Electroluminescent polyhedral oligomeric silsesquioxane-based nanoparticle. Chem. Mater..

[100] Froehlich J.D., Young R., Nakamura T., Ohmori Y., Li S., Mochizuki A., Lauters M., Jabbour G.E. (2007). Synthesis of multi-functional POSS emitters for OLED applications. Chem. Mater..

[101] Eom J.H., Mi D., Park M.J., Cho H.J., Lee J., Lee J.I., Chu H.Y., Shim H.K., Hwang D.H. (2009). Synthesis and properties of a polyhedral oligomeric silsesquioxane-based new light-emitting nanoparticle. J. Nanosci. Nanotechnol..

[102] Su X., Guang S., Li C., Xu H., Liu X., Wang X., Song Y. (2010). Molecular hybrid optical limiting materials from polyhedral oligomer silsequioxane: Preparation and relationship between molecular structure and properties. Macromolecules.

[103] Cheng C.C., Chu Y.L., Chu C.W., Lee D.J. (2016). Highly efficient organic-inorganic electroluminescence materials for solution-processed blue organic light-emitting diodes. J. Mater. Chem. C.

[104] Xiang K., Li Y., Xu C., Li S. (2016). POSS-based organic-inorganic hybrid nanomaterials: Aggregation-enhanced emission, and highly sensitive and selective detection of nitroaromatic explosives in aqueous media. J. Mater. Chem. C.

[105] Miniewicz A., Tomkowicz M., Karpinski P., Sznitko L., Mossety-Leszczak B., Dutkiewicz M. (2015). Light sensitive polymer obtained by dispersion of azo-functionalized POSS nanoparticles. Chem. Phys..

[106] Miniewicz A., Girones J., Karpinski P., Mossety-Leszczak B., Galina H., Dutkiewicz M. (2014). Photochromic and nonlinear optical properties of azo-functionalized POSS nanoparticles dispersed in nematic liquid crystals. J. Mater. Chem. C.

[107] Tkachenko I.M., Kobzar Y.L., Korolovych V.F., Stryutsky A.V., Matkovska L.K., Shevchenko V.V., Tsukruk V.V. (2018). Novel branched nanostructures based on polyhedral oligomeric silsesquioxanes and azobenzene dyes containing different spacers and isolation groups. J. Mater. Chem. C.

[108] Ledin P.A., Tkachenko I.M., Xu W., Choi I., Shevchenko V.V., Tsukruk V.V. (2014). Star-shaped molecules with polyhedral oligomeric silsesquioxane core and azobenzene dye arms. Langmuir.

[109] Su X., Guang S., Xu H., Yang J., Song Y. (2010). The preparation and optical limiting properties of POSS-based molecular hybrid functional materials. Dye. Pigment..

[110] Zhang Q.C., Xiao H., Zhang X., Xu L.J., Chen Z.N. (2019). Luminescent oligonuclear metal complexes and the use in organic light-emitting diodes. Coord. Chem. Rev..

[111] Chen K.-B., Chang Y.-P., Yang S.-H., Hsu C.-S. (2006). Novel dendritic light-emitting materials containing polyhedral oligomeric silsesquioxanes core. Thin Solid Films.

[112] Yang X., Froehlich J.D., Chae H.S., Li S., Mochizuki A., Jabbour G.E. (2009). Efficient Light-Emitting Devices Based on Phosphorescent Polyhedral Oligomeric Silsesquioxane Materials. Adv. Funct. Mater..

[113] Yang X., Froehlich J.D., Chae H.S., Harding B.T., Li S., Mochizuki A., Jabbour G.E. (2010). Efficient light-emitting devices based on platinum-complexes-anchored polyhedral oligomeric silsesquioxane materials. Chem. Mater..

[114] Yu T., Wang X., Su W., Zhang C., Zhao Y., Zhang H., Xu Z. (2015). Synthesis and photo- and electro-luminescent properties of Ir(III) complexes attached to polyhedral oligomeric silsesquioxane materials. RSC Adv..

[115] Zhao Y., Qiu X., Yu T., Shi Y., Zhang H., Xu Z., Li J. (2016). Synthesis and characterization of 8-hydroxyquinolinolato-iridium(III) complex grafted on polyhedral oligomeric silsesquioxane core. Inorganica Chim. Acta.

[116] Yu T., Xu Z., Su W., Zhao Y., Zhang H., Bao Y. (2016). Highly efficient phosphorescent materials based on Ir(III) complexes-grafted on a polyhedral oligomeric silsesquioxane core. Dalt. Trans..

[117] Kolb H.C., Finn M.G., Sharpless K.B. (2001). Click Chemistry: Diverse Chemical Function from a Few Good Reactions. Angew. Chemie Int. Ed..

[118] Li Y., Dong X.H., Zou Y., Wang Z., Yue K., Huang M., Liu H., Feng X., Lin Z., Zhang W. (2017). Polyhedral oligomeric silsesquioxane meets “click” chemistry: Rational design and facile preparation of functional hybrid materials. Polymer.

[119] Huisgen R. (1963). 1,3-Dipolar Cycloadditions. Past and Future. Angew. Chemie Int. Ed. Engl..

[120] Ervithayasuporn V., Kwanplod K., Boonmak J., Youngme S., Sangtrirutnugul P. (2015). Homogeneous and heterogeneous catalysts of organopalladium functionalized-polyhedral oligomeric silsesquioxanes for Suzuki–Miyaura reaction. J. Catal..

[121] Ervithayasuporn V., Abe J., Wang X., Matsushima T., Murata H. (2010). Synthesis, characterization, and OLED application of oligo (p-phenylene ethynylene) s with polyhedral oligomeric silsesquioxanes (POSS) as pendant groups. Tetrahedron.

[122] Zhao G., Zhu Y., Guang S., Ke F., Xu H. (2018). Facile preparation and investigation of the properties of single molecular POSS-based white-light-emitting hybrid materials using click chemistry. New J. Chem..

[123] Zhu Y.K., Guang S.Y., Xu H.Y. (2012). A versatile nanobuilding precursor for the effective architecture of well-defined organic/inorganic hybrid via click chemistry. Chin. Chem. Lett..

[124] Costela Á., López-Arbeloa Í., García-Moreno I., Trastoy B., Bañuelos J., Chiara J.L., Pérez-Ojeda M.E. (2011). Click Assembly of Dye-Functionalized Octasilsesquioxanes for Highly Efficient and Photostable Photonic Systems. Chem. A Eur. J..

[125] Tutov M.V., Sergeev A.A., Zadorozhny P.A., Bratskaya S.Y., Mironenko A.Y. (2018). Dendrimeric rhodamine based fluorescent probe for selective detection of Au. Sens. Actuators B Chem..

[126] Lin M., Luo C., Xing G., Chen L., Ling Q. (2017). Influence of polyhedral oligomeric silsesquioxanes (POSS) on the luminescence properties of non-conjugated copolymers based on iridium complex and carbazole units. RSC Adv..

